# In Vivo and In Vitro Characterization of Primary Human Liver Macrophages and Their Inflammatory State

**DOI:** 10.3390/biomedicines9040406

**Published:** 2021-04-09

**Authors:** Andrea Zimmermann, René Hänsel, Kilian Gemünden, Victoria Kegel-Hübner, Jonas Babel, Hendrik Bläker, Madlen Matz-Soja, Daniel Seehofer, Georg Damm

**Affiliations:** 1 Department of Hepatobiliary Surgery and Visceral Transplantation, University Hospital, Leipzig University, 04103 Leipzig, Germany; andrea.lohrenz@sikt.uni-leipzig.de (A.Z.); rene.haensel@sikt.uni-leipzig.de (R.H.); k.gemuenden@gmx.net (K.G.); victoria.kegel@yahoo.de (V.K.-H.); jonas.babel@medizin.uni-leipzig.de (J.B.); daniel.seehofer@medizin.uni-leipzig.de (D.S.); 2 Saxonian Incubator for Clinical Translation (SIKT), Leipzig University, 04103 Leipzig, Germany; 3 Institute for Medical Informatics, Statistics and Epidemiology (IMISE), Leipzig University, 04107 Leipzig, Germany; 4 Institute for Pathology, University Hospital, Leipzig University, 04103 Leipzig, Germany; hendrik.blaeker@medizin.uni-leipzig.de; 5 Rudolf-Schönheimer-Institute of Biochemistry, Leipzig University, 04103 Leipzig, Germany; madlen.matz-soja@medizin.uni-leipzig.de; 6 Department for Hepatology, University Hospital, Leipzig University, 04103 Leipzig, Germany

**Keywords:** human liver, liver cell isolation, primary human liver cells, hepatic macrophages, Kupffer cells, monocyte-derived macrophages, macrophage heterogeneity, inflammatory liver diseases, in vitro liver model

## Abstract

Liver macrophages (LMs) play a central role in acute and chronic liver pathologies. Investigation of these processes in humans as well as the development of diagnostic tools and new therapeutic strategies require in vitro models that closely resemble the in vivo situation. In our study, we sought to gain further insight into the role of LMs in different liver pathologies and into their characteristics after isolation from liver tissue. For this purpose, LMs were characterized in human liver tissue sections using immunohistochemistry and bioinformatic image analysis. Isolated cells were characterized in suspension using FACS analyses and in culture using immunofluorescence staining and laser scanning microscopy as well as functional assays. The majority of our investigated liver tissues were characterized by anti-inflammatory LMs which showed a homogeneous distribution and increased cell numbers in correlation with chronic liver injuries. In contrast, pro-inflammatory LMs appeared as temporary and locally restricted reactions. Detailed characterization of isolated macrophages revealed a complex disease dependent pattern of LMs consisting of pro- and anti-inflammatory macrophages of different origins, regulatory macrophages and monocytes. Our study showed that in most cases the macrophage pattern can be transferred in adherent cultures. The observed exceptions were restricted to LMs with pro-inflammatory characteristics.

## 1. Introduction

The liver exerts a unique capacity for regeneration after extensive tissue damage [1]. Here, hepatic macrophages are the first responders to tissue damage and orchestrate the immunological reactions leading to tissue repair [2]. Liver macrophages (LMs) are mainly composed of liver-resident phagocytes named Kupffer cells (KCs) [3]. It is known from mouse experiments that KCs are not the only macrophage population in the liver, containing also bone marrow monocyte-derived macrophages (MDMs), capsular macrophages, and recruited peritoneal macrophages inherited from previous inflammatory events [4], which could also be shown similarly in human livers [5]. KCs and MDMs are the best investigated from these populations and play a major part in health and disease. Both populations are positive for the macrophage marker CD68 and cannot be distinguished further by origin specific markers in humans. In healthy livers, resident KCs dominate the hepatic macrophage pool and represent 80–90% of all tissue macrophages in the entire body [6]. In homeostasis, KCs line the walls of liver sinusoids and perform essential functions, such as the phagocytosis of pathogens and particles, as well as the first response following liver injuries [2].

MDMs are derived from circulating monocytes that are recruited to the liver by active toll-like receptor (TLR) signaling from immune-sensing cells and a subsequent increase in chemokines as a consequence of injuries. Following liver infiltration, monocytes become activated depending on the environment and accumulate at the site of the injury. At steady state, they were reported to have a short half-life of two days [3]. Initially, MDMs show predominantly pro-inflammatory characteristics but can also differentiate into anti-inflammatory macrophages in chronic tissue damage [7].

Activated macrophages, including KCs and MDMs, can be grouped in a simplified way as pro-inflammatory M1 (classically activated) and anti-inflammatory M2 (alternatively activated) macrophages [8,9,10]. Their dominant appearance is caused by the fact that M1 and M2 markers can be expected to be present on the same cell based on the theory of overlapping activation types, which in turn results in a wide spectrum of macrophage subsets [9,10,11]. The pro-inflammatory M1 phenotype is activated by signals such as microbial LPS or cytokines such as interferon-γ (IFN-γ) and tumor necrosis factor-α (TNF-α). As a result, they produce pro-inflammatory mediators such as interleukin (IL)-1, IL-6, IL-12, or TNF-α as well as significantly increased amounts of reactive oxygen species (ROS) and nitric oxide (NO) which are collectively known as reactive oxygen intermediates (ROIs). Second, the anti-inflammatory M2 phenotype is activated by immunomodulatory signals such as IL-4, IL-10 and IL-13, which produce anti-inflammatory and immunoregulatory cytokines such as IL-10, TGF-β and prostaglandin E2 (PGE2) [12]. The expression of various markers on differentially activated macrophages has already been investigated, identifying CD80 and CD86 for M1 macrophages as well as CD206 and CD163 for M2 macrophages [13,14].

Because of their plasticity and importance in various inflammation processes, therapeutic strategies for the treatment of liver diseases are increasingly targeting macrophages. Starting points for such interventions offer for example the modeling of KC activation, monocyte recruitment to the liver or macrophage polarization and differentiation [15]. However, the use of macrophages in in vitro culture systems to investigate processes such as particle clearance and drug-induced liver injury (DILI) or in long-term co-cultures with hepatocytes for maintaining cellular homeostasis are possible applications. In contrast, frequently used mouse models are not fully representative of human diseases even if the macrophage heterogeneity is better understood in mice than in humans [15]. To use primary LMs for applications and functional diagnostics, the cells have to be isolated. Thus, questions arise regarding whether isolated macrophages in in vitro culture systems reveal the same functionalities and activation states as those in vivo.

Therefore, the aim of the present study was to investigate the inflammatory states and functions of primary human LMs both in vivo and in vitro. For this purpose, hepatic macrophages were characterized in vivo and in vitro after cell isolation. Our results show that the characteristics of isolated LMs match both the inflammatory state of the donor pathology and the tissue characteristics. In addition, the inflammatory state of adherent macrophage cultures corresponds in majority to the in vivo situation, allowing the usage of these cultures for human in vitro liver models.

## 2. Materials and Methods

### 2.1. Media and Chemicals

The LM culture medium was based on RPMI 1640 with phenol red, low endotoxin and without L-Glutamine supplemented with 10% FCS, 20 mM L-Glutamine, and 1000 U/ml Penicillin/1000 µg/ml Streptomycin (both by Thermo Fisher Scientific, Waltham, MA, USA). The LM starvation medium contained the same supplements like LM culture medium but no FCS.

Phosphate-buffered saline supplemented with calcium and magnesium ions (PBS) was purchased from Thermo Fisher Scientific (Waltham, MA, USA). EasyColl and Hanks balanced salt solution containing calcium and magnesium ions (HBSS) were provided by Biochrom (Berlin, Germany). All other chemicals were purchased from Sigma (St. Louis, MO, USA) if not stated differently.

### 2.2. Cell Culture

All media, solutions and materials were sterilized by filtration or by autoclaving. Every cell culture work was performed under laminar flow cabinets and all cell cultures were performed in CO_2_ incubators at a temperature of 37 °C, an atmosphere of 5% CO_2_ and 95% humidity. The cell cultures were checked regularly, and microscopic pictures were taken for quality control (Eclipse TS100, Nikon, Tokyo, Japan).

### 2.3. Tissue Samples

Liver tissue samples were obtained from macroscopically “healthy” tissue that remained from resected human liver of patients with primary or secondary liver tumors or benign local liver diseases. Informed consent of the patients for the use of tissue for research purposes was obtained according to the ethical guidelines of Leipzig University Hospital (177/16-lk, 12 July 2016). Liver tissue sample were collected for immunohistochemical analyses (Table 1 and Table S1) or liver cell isolations (Table 2).

### 2.4. Immunohistochemical Staining of Paraffin Embedded Tissue

Immunohistochemistry (IHC) allows the determination and visualization of the localization of the proteins in the intact tissue. For investigation of the tissue distribution of hepatic macrophages, the human liver tissue samples (Table 1) were fixed with paraformaldehyde (PFA), embedded in paraffin, sectioned into 3.5 µm thick slices using a microtome (Microm HM 355S, Thermo Fisher Scientific, Waltham, USA), and mounted on slides.

Preparation for the antibody staining started with the rehydration of the tissue before a heat induced epitope retrieval was performed using a citric buffer (pH 6.0). After that, endogenous peroxidases were blocked by washing for 15 min in Tris-buffered saline (TBS) supplemented with 3% H_2_O_2_ and tissue was permeabilized by treating three times for 10 min with 0.2% Triton X-100 added to TBS. Afterwards, slices were blocked for unspecific bindings by an incubation with 10% bovine serum albumin (BSA) and 0.3% Triton X-100 in TBS for 45 min at room temperature (RT). For detection of LMs and LM subpopulations, specific primary antibodies were used (Table 3). All antibodies were diluted in TBS supplemented with 1% BSA and 0.03% Triton X-100 and incubated at 4 °C in a humid chamber overnight. The following day, slices were washed in TBST (TBS + 0.5% Tween20) before detection. The EnVision+Dual Link System-HRP (Dako, Glostup, Denmark) was used according to manufacturer instructions for a peroxidase staining of the primary antibodies against CD68 and the subsequent visualization. The antibodies against the targets CD80 and CD163 were visualized using Peroxidase-conjugated secondary antibodies (Table 3). For detection of peroxidase activity 3,3′-Diaminobenzidine (DAB) was used as chromogen. All reactions were stopped using TBS and cell nuclei were counterstained with hematoxylin. Finally, the slices were dehydrated in ethyl alcohol in various concentrations as well as in xylene before they were embedded using the non-aqueous mounting medium Entellan. Negative controls were made from all donors and treated in the same way but without usage of a primary antibody. In addition, a Hematoxylin and Eosin (H&E) staining was performed to evaluate the tissue structure.

### 2.5. Image Analysis of Immunohistochemical Stainings

Whole slide images were captured in transmitted light mode using a Slide Scanner (AxioScan Z1, Carl Zeiss, Oberkochen, Germany) with a 20×/0.5 Plan-Apochromat objective and stored as raw data in the Carl Zeiss proprietary image pyramid format (CZI) with an object-related nominal pixel size of 0.221 µm × 0.221 µm. The images represent whole tissue sections of the immunohistochemical stainings for CD68, CD163, and CD80, as well as controls. The whole tissue scans of all donors and immunohistochemical stainings are accessible by using the platform ‘LiSyM SEEK’ (https://seek.lisym.org/investigations/25 (accessed on 8 April 2021)).

For further pre-processing of the image raw data the Zeiss Zen software was used [16]. Pre-processing included a contrast enhancement by tonal value spreading and a visually controlled adjustment of the transformation characteristic of the RGB histogram. Further preparation for image analysis included the storage of the raw data of each donor manually in a lossless mode for 8-bit Tagged Image File Format (TIFF) and the division into 1000 × 1000 px tiles. In summary, the total number of image tiles for 15 donors was 88,583 for CD68, 83,447 for CD163, and 76,215 for CD80.

**Figure 1:**
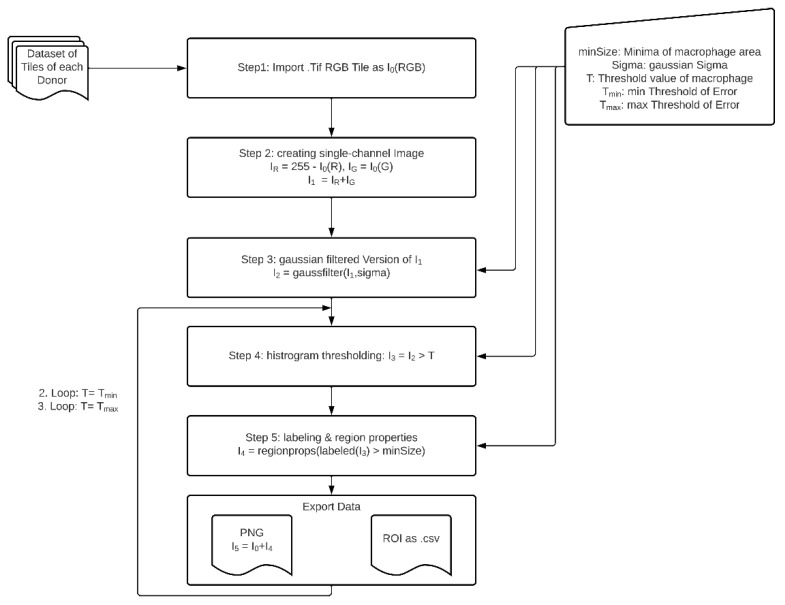
Flowchart of macrophage segmentation method. Human liver tissue samples were stained with HRP-conjugated antibodies against macrophages and visualization was done using DAB. Images were taken of whole tissue sections and further processed in several steps to finally identify, label, and analyze the regions corresponding to stained macrophages.

The method described here (Figure 1) exploits the RGB property of the macrophage specific staining using DAB resulting in a brown appearance. The blue channel values of this brown color in the RGB profile are low. Therefore, a high intensity signal was obtained from the remaining red and the green channel. After image import (Step 1), each tile I_0_ was separated into its color channels red (I_0_(R)) and green (I_0_(G)). To generate a segmentable high intensity signal I_1_, the inverted red channel I_R_, and the green channel I_G_ are computed as linear combination (Step 2).

$$I_{R}=255-I_{0}\left(R\right)$$

$$I_{G}=I_{0}\left(G\right)$$

$$I_{1}=I_{R}+I_{G}$$

$$I_{1}=\left(255-I_{0}\left(R\right)\right)+I_{0}\left(G\right)$$

To minimize the significant amount of noise of the image by preserving the image structure we decided to create a Gaussian filtered version *I*_2_ of *I*_1_ with a sigma width of 5 px (Step 3). The sigma width was repeatedly determined experimentally, taking care to preserve the original edge structure of the features. Then *I*_2_ is binarized with defined criterion *T* for thresholding and results in a black and white feature mask *I*_3_ (Step 4) and is obtained as

$$I_{3}=\;\{\begin{array}{c} 1,\;for\;I_{2}\geq T\\ 0\;for\;I_{2}<T. \end{array}$$

In the next step the generated regions of interest corresponding to the white pixels of the images are labelled and these feature regions are analyzed according to geometrical specifications *I*_4_ (Step 5). In two further loops, a threshold window (T_min_ and T_max_) around the base threshold *T* as well as corresponding label and region data were generated as described in Step 4 and Step 5.

**Figure 2:**
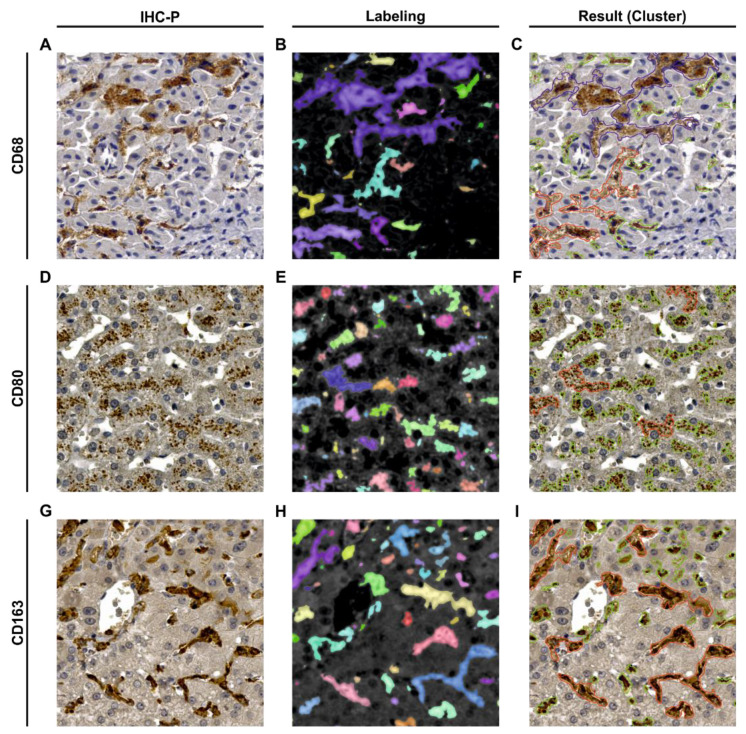
Visualization of the input, the intermediate step of labeling, and the output of the macrophage segmentation method. The first column of images (**A**,**D**,**G**) corresponds to original RGB images. The middle column of images (**B**,**E**,**H**) show gaussian filtered versions of the output mask I4 of labeled feature regions. Here, the different colors represent the resulting individual objects consisting of labeled pixels which in turn correspond to the macrophages. The third column of images (**C**,**F**,**I**) visualizes the clustered feature regions (green: Cluster 1 from 10–300 µm^2^; red: Cluster 2 from 300.01–2000 µm^2^; blue: Cluster 3 with >2000.01 µm^2^). **A**–**C**: Donor D09 (Tile: s1_m0127); **D**–**F**: Donor D01 (Tile: m137); **G**–**I**: Donor D06 (Tile: s2_m0697).

As input for the method described above, a three-channel RGB image in TIFF with a depth of 8-bit per channel was required (Figure 2A,D,G). The output of the method per image tile included a Comma Separated Values (CSV) file exporting the data of the feature regions of all three fixed thresholds *T*, T_min_ and T_max_. The data consisted of a list of numbered features and the corresponding measured areas. For a subjective evaluation of the results, a visualization of the segmented feature regions in the original image I_0_ was saved as a Portable Network Graphic (PNG) (Figure 2B,E,H).

The method was finally implemented using the image processing package Fiji, a software package for bio image processing based on ImageJ [17]. To be able to analyze all tissue images automatically and iteratively, the ImageJ procedures were implemented in ImageJ’s own script language.

The base threshold values were different for the three specific immunohistochemical stainings and determined experimentally using a couple of self-selected significant image tiles by applying the Yen method based on a maximum correlation criterion for bilevel thresholding in the histogram [18]. This process resulted in base threshold values of *T* = 13, *T* = 18, and *T* = 21 for CD68, CD80, and CD163, respectively.

In order to be able to statistically assess the robustness of the method we decided to set a threshold window around the base threshold named T_min_ and T_max_. Due to the characteristic shape of the histogram data, we experimentally set T_max_ to four greyscale values above and T_min_ to three greyscale values below the base threshold *T*. The geometric parameters of macrophages for Step 5 were determined experimentally using a couple of self-selected significant image tiles and thus each feature region >10 µm^2^ was segmented as a macrophage.

The data analysis of the individual CSV files were implemented in Python. A random examination of the results showed the necessity to divide the data into three clusters (Figure 2C,F,I). Cluster 1 describes feature regions with an area between 10 to 300 µm^2^ representing single macrophages and small groups of macrophage, whereas Cluster 2 describes feature regions with an area between 300.01 to 2000 µm^2^ representing larger groups of macrophages. Cluster 3 describes feature regions that are >2000 µm^2^ traced to unspecific stainings. Therefore, Cluster 3 was not considered for further analyses.

### 2.6. Isolation of Hepatic Macrophages

Human liver cells composed of primary human hepatocytes (PHH) and non-parenchymal cells (NPCs) including hepatic macrophages were isolated from liver tissue samples (Table 2) by a two-step ethylenediaminetetraacetic acid (EDTA)/collagenase perfusion technique as described elsewhere [19]. A modification was done by adding glucose and insulin to the Perfusion solution I as described by Damm et al. [20]. In brief, PHH were separated from the NPCs and the latter were cleaned up by a density gradient centrifugation step. The macrophage population was separated from the remaining NPCs fraction by their ability to adhere in a very short time. Therefore, the NPCs fraction was seeded on 24-well plates (Greiner, Kremsmünster, Austria) for XTT and DCF assay and 4-well Chamber-Slides (ibidi, Martinsried, Germany) for immunofluorescence staining in a density of 2 × 10^6^ macrophages per ml using an adherence time of 20 min at 37 °C. Then, remaining NPCs were removed by washing twice with warm HBSS.

### 2.7. XTT Viability Assay

The Cell Proliferation Kit II (Roche, Basel, Switzerland) was used to evaluate cell activities. During this colorimetric assay, the yellow tetrazolium salt 2,3-Bis-(2-Methoxy-4-Nitro-5-Sulfophenyl)-2H-Tetrazolium-5-Carboxanilide (XTT) is reduced to a highly colored and water-soluble formazan dye by dehydrogenase enzymes in the presence of an electron-coupling reagent. This conversion only occurs in metabolically active cells and thus, the amount of the formazan produced can correlate to viable cells in the sample.

The assay was performed according to the manufacturer’s protocol and XTT labeling mixture was added to the starvation medium described above. After cells were washed, this working solution was added to each well and cells were incubated for 2 h at 37 °C in a CO_2_ incubator. Finally, 100 µl of these solutions were transferred to 96-well plates and the absorbance was quantified by measuring the absorbance at a wavelength of 450 nm using a microplate reader (Synergy H1, BioTek Instruments, Winooski, VT, USA).

### 2.8. DCF Assay for ROI Detection

ROIs are produced by macrophages as a part of inflammatory immune reactions but play also an essential role in intracellular signaling pathways. The formation of intracellular ROIs was measured quantitatively by using the fluorogenic dye 2,7–dichlorofluorescein diacetate (DCF-DA). After diffusion into a cell, DCF-DA is initially deacetylated by cellular esterases to a non-fluorescent compound which is then oxidized by ROIs into the highly fluorescent compound 2,7–dichlorofluorescein (DCF).

The assay was performed like described elsewhere [21]. In brief, cells were incubated in a working solution containing 20 µM DCF-DA for 30 min at 37 °C in a CO_2_ incubator. Subsequently, this solution was aspirated and directly replaced by RPMI without serum and phenol red and without performing a washing step. Then, cells were incubated for further 60 min at 37 °C before 100 µL of these solutions were transferred to 96-well plates. Fluorescence was measured at an excitation wavelength of 480 nm and an emission wavelength of 530 nm using a microplate reader.

### 2.9. Determination of Protein Content

The stable and water-soluble sodium salt bicinchoninic acid (BCA) is used to determinate the protein content of cells. This assay takes advantage of the fact that double positively charged copper ions (Cu^2+^) from copper(II) sulfate (CuSO_4_) in an alkaline environment are reduced by proteins to Cu^+^ forming violet-colored complexes with BCA. The color produced from this reaction is proportional to the protein content.

For the BCA assay, cells were first disrupted using a lysis buffer consisting of PBS (w/o) supplemented with 0.1% sodium dodecyl sulfate (SDS), 0.5% Triton X-100, and 50 mM Trizma HCl. Then, 20 µL per resulting protein sample were added in triplicates to 96-well plates and mixed with 300 µL working solution consisting of BCA supplemented with CuSO_4_ in a 1:50 ratio. The plates were incubated for 30 min at 37 °C in a CO_2_ incubator each containing a standard curve using BSA dissolved in water. The protein contents were finally quantified by measuring the absorption of the produced colored complexes at a wavelength of 550 nm using a microplate reader.

### 2.10. Immunofluorescence Staining

LM characterization was performed in cell suspensions as well as with cells in adherent cultures using immunofluorescence staining. For investigation of cell suspension flow cytometry and for cultured cells laser scanning microscopy was used, respectively. For both methods, previously macrophages were fixed with 10X Zinc Fixative (BD Biosciences, Franklin Lakes, NJ, USA) at 4 °C overnight. The following day, cells were washed with staining buffer consisting of PBS supplemented with 0.1% BSA and 2 mM EDTA and treated with human Fc Block (BD Biosciences) for 10 min at RT to prevent unspecific bindings. A permeabilization step was also done for both methods, using lysis buffer consisting of staining buffer supplemented with 0.5% Tween20 to allow staining of intracellular epitopes. Directly labeled antibodies were used to identify macrophages and their respective subpopulations (Table 4).

#### 2.10.1. Cell Staining for Flow Cytometry

Solutions used for the staining were cooled down to 4 °C, centrifugation steps were also performed at 4 °C, and after the addition of the antibodies all working steps were realized protected from light.

First after Zinc fixation, cell suspensions were centrifuged at 650× *g* for 5 min and cell pellets were treated with human Fc Block. For extracellular staining, cells were incubated with directly labeled antibodies for 10 min at 4 °C (Table 4). For the subsequent intracellular staining, cells were permeabilized, centrifuged at 650× *g* for 5 min and incubated with directly labeled antibodies diluted in lysis buffer for 10 min (Table 4). After antibody staining, macrophages were washed with staining buffer and finally the cells were resuspended again in the same buffer in order to be subsequently measured on the flow cytometer (FACS Canto II, BD Biosciences). Autofluorescence controls were treated as described above without using antibodies and FMO (fluorescence minus one) controls were performed for all fluorophores.

For the viability staining, unfixed cells were centrifuged at 650× *g* for 5 min and pellets were incubated with eFluor780 staining buffer for 30 min at 4 °C. Afterwards, cells were washed using staining buffer and fixed over night at 4 °C using Zink fixative. Next day, cells were washed with staining buffer and finally cells were resuspended again in the same buffer in order to be subsequently measured on the flow cytometer. An autofluorescence control and a mixture of living and dead cells were used as staining controls.

For the staining with the compensation beads antibodies were placed in a tube containing FACSFlow, then anti-REA or anti-mouse Igκ beads were added and samples were incubated for 5–10 min in the dark at RT. Finally, FACSFlow was added and the compensation setup was done according to the recommendations of the manufacturer.

Adherent hepatic macrophages were used to establish the gating strategy as shown in Figure S1 and this process was applied for all further experiments using FlowJo software. Therefore, the individual gates were placed using the corresponding FMO control. After gating the cell population of interest and from this the single cells, two CD68+ subpopulations with different FSC due to different sizes were first identified. These populations were analyzed separately for their expression of CD86 and CD206 (data not shown) and subsequently summarized again for the results presented here. In the histograms, the cell number was normalized to the modal value.

#### 2.10.2. Cell Staining of Adherent Cells

First after zinc fixation, adherent macrophages were treated with human Fc Block and then incubated with directly labeled antibodies for 10 min at 4 °C for extracellular stainings (Table 4). For the subsequent intracellular staining, cells were washed in staining buffer, permeabilized using lysis buffer and incubated with directly labeled antibodies diluted in lysis buffer for 10 min at RT (Table 4). After antibody staining, macrophages were washed with staining buffer, cell nuclei were stained using 1 µg/ml Hoechst 33342 for 5 min at RT and cells were washed twice again before the slides were filled with Mounting Medium (ibidi). Autofluorescence controls were treated as described above without using antibodies. The completed staining was investigated using a laser scanning microscope (LSM700, Carl Zeiss).

### 2.11. Statistical Analysis

Statistical analyses and chart design were performed using GraphPad Prism 7. Data are plotted as mean values ± standard deviation as well as relative frequencies and multiples of the control group. Furthermore, data were analyzed by one-way analysis of variance (ANOVA) followed by a Bonferroni (comparison of different treatments) post-hoc test. Differences were considered as significant if the resulting *p*-value adjusted by the Bonferroni method was less than 0.5 (*p* ≤ 0.05 (*), *p* ≤ 0.01 (**), *p* ≤ 0.001 (***), *p* ≤ 0.0001 (****)).

## 3. Results

### 3.1. Human Liver Tissue Sections Showed Disease Specific Patterns of Lobular Macrophage Reactivity

For creation of an in vivo reference we evaluated the activity of hepatic macrophages in human liver tissue sections using immunohistochemical staining. The whole LM population was stained using an antibody against CD68 whereas antibodies against CD80 and CD163 were used to identify pro- and anti-inflammatory macrophages, respectively. In summary, liver tissue samples from 15 individual donors with different underlying diseases were investigated. The underlying diseases were divided into four groups: 1. hepatocellular carcinoma (HCC) and 2. cholangiocarcinoma (CCA), including Klatskin tumors (perihilar cholangiocarcinoma (pCCA)) representing primary liver tumors, 3. colorectal liver metastasis (CRLM) representing secondary tumors, and 4. benign liver tumors. Those donors with benign diseases, together with patients with secondary tumors without chemotherapy and hepatitis, served as a control group with relatively “healthy” liver tissue (Table 1).

Clinical data of the patients (Table S1) revealed elevated levels of C-reactive protein (CRP), ranging from 20.6 to 38.7 mg/L in five patients (D04, D07, D09, D11, and D12). However, liver enzymes of all patients indicated no signs of acute liver damage. Instead, for a significant proportion of patients, histopathological investigations showed slight lipid accumulation of approximately 5% as well as slight to moderate portal inflammation together with at least portal fibrosis and thus chronic liver damage (Table 1). Furthermore, two donors (D10 and D11) had Child A cirrhosis. Additionally, the liver tissues from four patients (D03, D04, D10, and D14) showed increased levels of lipid accumulation. All patients diagnosed with CCA (D04 and D06–D08) had signs of cholestasis, which was also present in one patient with chronic cholangitis (D07).

Taken together, the clinical data revealed no signs of any acute inflammatory event. However, all tissue samples, including those from the control group, showed at least slight chronic pathological changes indicating slight to moderate chronic inflammation.

**Figure 3:**
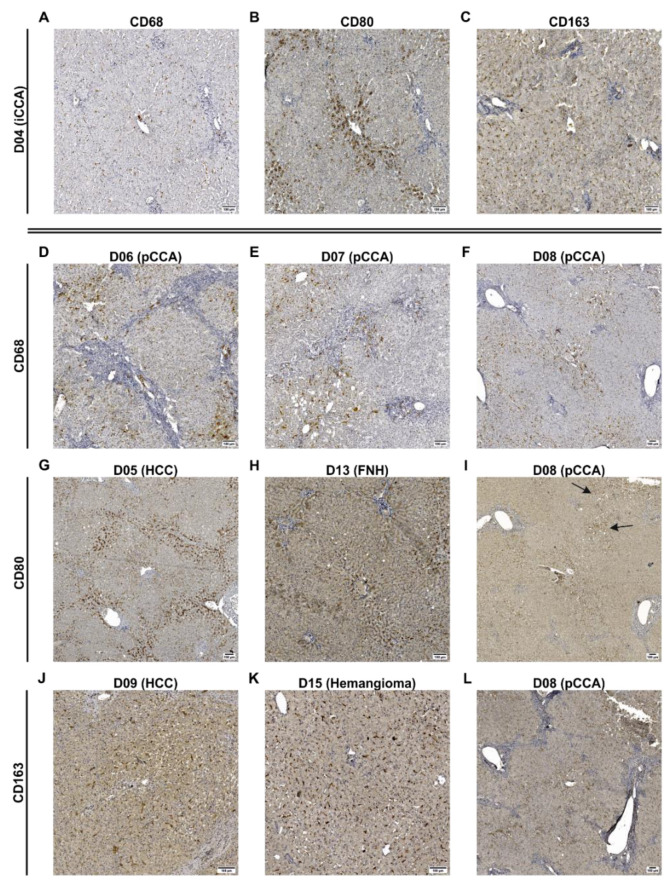
Determination of spatial distribution of different populations of macrophages in human liver tissue sections. Human tissue samples (detailed donor data: Table 1 and Table S1) were investigated by immunohistochemical stainings for general macrophages (CD68), pro-inflammatory (CD80), and anti-inflammatory macrophages (CD163). Detection was performed using HRP-coupled secondary antibodies and visualization was done using DAB. Representative pictures of these are shown here for illustration purposes. (**A**–**C**) Images originate from a patient with iCCA (D04) showing stainings for CD68, CD80 and CD163 of the same tissue sections. (**D**–**F**) Images show stainings for CD68 of three donors with pCCA (D06, D07 and D08) indicating correlations between CD68+ cells and fibrosis. (**G**–**I**) Images show stainings for CD80 of three different donors accumulating around central veins or portal fields. The arrows in (**I**) indicate CD80+ cells correlating with lipid accumulations. (**J**–**L**) Images show stainings for CD163 of three different donors showing evenly distributed macrophages. Images were taken using a Slide Scanner (AxioScan Z1, Carl Zeiss, Oberkochen, Germany) at 20× magnification. The whole tissue scans of all donors and immunohistochemical stainings are accessible by using the platform ‘LiSyM SEEK’ (https://seek.lisym.org/investigations/25 (accessed on 8 April 2021)). (FNH: focal nodular hyperplasia, HCC: hepatocellular carcinoma, iCCA: intrahepatic cholangiocarcinoma, pCCA: perihilar cholangiocarcinoma).

Qualitative investigation of the stained tissue sections revealed that staining for CD68 and CD163 resulted in complete labeling of the macrophages, while CD80 staining appeared more diffuse and granular. Both inflammatory markers showed a correlation with CD68, indicating the presence of inflammatory shaped subpopulations of LMs (Figure 3A–C). The occurrences of both CD80+ and CD163+ cells without correlations with CD68+ were also confirmed by analyzing the same regions in liver tissue sections of a donor on parallel with these markers. (Figure S2).

The investigation of different liver pathologies revealed a striking correlation of the macrophage number with the degree of portal fibrosis in liver tissue samples from patients with Klatskin tumors (pCCA; Figure 3D–F, Table 1). One patient diagnosed with iCCA (D04) showed an increased number of CD80+ cells that accumulated around the central vein (Figure 3B), whereas in another patient with HCC (D05), these pro-inflammatory cells accumulated around the portal fields and lobular boundaries (Figure 3G). Additionally, CD80+ cells were locally confined in spots correlating with unregularly lipid accumulations (D08, Figure 3I). In general, CD163+ cells (Figure 3J–L) were more homogeneously distributed in the liver lobule than CD80+ cells (Figure 3G–I). Most samples showed a high accumulation of CD163 in intrasinusoidal KCs with clear positive staining (D09), whereas one patient with pCCA (D08) presented an accumulation around the central vein, showing more granular staining as typically observed only for CD80.

### 3.2. Bioinformatic Analysis Revealed That Human Liver Macrophages In Vivo Are Characterized by a Predominantly Anti-Inflammatory Activation State

For quantitative investigation of LMs, whole slides of the stained tissue sections were scanned, and the cell areas were quantified using an automated imaging analysis method. The macrophage quantification was done by normalization of the cell area to the whole tissue section area. LM detection was clustered by size into two groups: group one represented solitary macrophages and small clusters of macrophages (Cluster 1), and group two represented larger clusters of macrophages (Cluster 2). In order to allow statistical analyses regarding the differences of the donors (ANOVA followed by Bonferroni correction), patients with the same diseases were grouped, whereby donors with CCA also included patients with pCCA.

**Figure 4:**
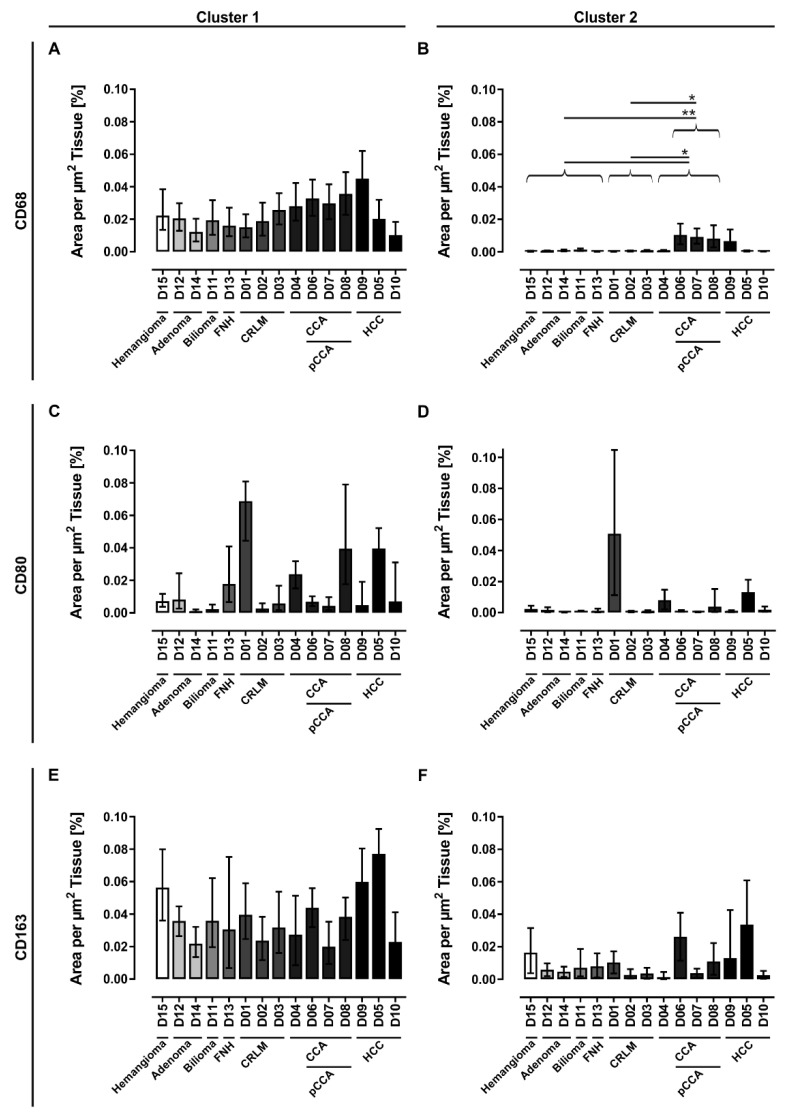
Quantification of different populations of macrophages in human liver tissue sections. Human tissue samples were investigated by immunohistochemical staining for (**A**,**B**) general macrophages (CD68), (**C**,**D**) pro-inflammatory (CD80) and (**E**,**F**) anti-inflammatory macrophages (CD163). An automated imaging analysis method was used to identify the cell areas that were normalized to the whole section area. Additionally, the macrophage areas are clustered by size whereas (**A**,**C**,**E**) Cluster 1 (10–300 µm^2^) represents solitary macrophages and small clusters of macrophages and (**B**,**D**,**F**) Cluster 2 (300.01–2000 µm^2^) represents larger clusters of macrophages. The error bars represent the error of the detection method setting a threshold window around the base threshold that was used for cell segmentation. For determination of significantly differences, donors were grouped according their disease pattern (detailed donor data: Table 1 and Table S1). *: *p* ≤ 0.05, **: *p* ≤ 0.01. (CCA: cholangiocarcinoma, CRLM: colorectal liver metastasis, FNH: focal nodular hyperplasia, HCC: hepatocellular carcinoma, pCCA: perihilar cholangiocarcinoma).

The analyses revealed that benign liver diseases and secondary tumors tend to show lower numbers of CD68+ cells. In contrast, primary liver tumors are a heterogeneous group with single examples of low macrophage numbers, but the majority show high numbers of CD68+ cells. Larger clusters of CD68+ cells detected in Cluster 2 were restricted to primary liver tumors and were in case of CCA and pCCA significantly different in comparison to the control group (Control vs. CCA: *p* = 0.0265, Control vs. pCCA: *p* = 0.0057) and the group of secondary liver tumors (CRLM vs. CCA: *p* = 0.0489, CRLM vs. pCCA: *p* = 0.0109). These patients with CCA were also striking in Cluster 1 as they tended to show higher LM numbers compared to the control group and the group of secondary liver tumors (Figure 4A).

In general, the characterization of inflammatory subpopulations revealed LMs with predominantly anti-inflammatory characteristics. Highest numbers of CD163+ cells were detected in liver samples from patients diagnosed with HCC (D05 and D09) and in tissue sample from patient D15 with hemangioma (Figure 4E). CD80+ cells were found in higher numbers only in some single tissue samples, including tissue from a patient with CRLM (D01), two patients with CCA (D04 and D08), and one HCC patient (D05, Figure 4C). In addition, this characterization of inflammatory subpopulations showed that anti-inflammatory macrophages tend to build larger clusters than pro-inflammatory macrophages (Figure 4C–F).

Taken together, our tissue analyses revealed that patients with primary liver tumors show the highest numbers of CD68+ macrophages both as solitary cells and in clusters. In addition, the quantitative clustering analysis revealed that the majority of LMs were solitary and that LMs from all donors are characterized by a predominantly anti-inflammatory activation state. Our qualitative analysis revealed that these CD163+ cells were evenly distributed over the whole liver lobule with a tendency to build locally restricted accumulations. In contrast, high pro-inflammatory activation states are rare, and CD80+ cells can be found predominantly as solitary cells around the central and portal veins.

### 3.3. Isolation of Primary Human Liver Macrophages Results in Cell Suspensions and Cultures of Varying Quality and Quantity

**Figure 5:**
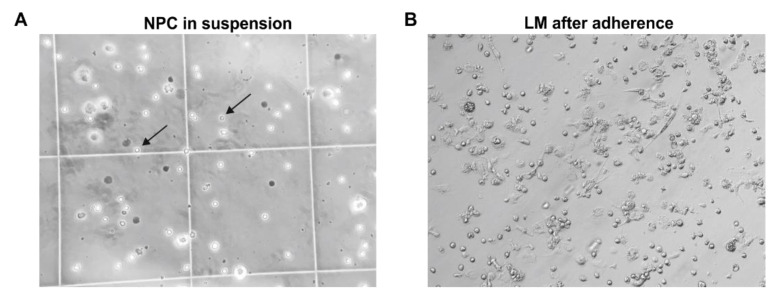
Microscopic images of isolated hepatic NPCs. (**A**) NPCs in suspension including cells with Kupffer cell-like morphology (arrows). (**B**) Human LM after adherence. Images were taken with an inverted light microscope at 20× magnification. (LMs: liver macrophages, NPCs: non-parenchymal cells).

Primary hepatic NPCs were isolated from 18 donors (Table 2) using a two-step EGTA/collagenase perfusion technique. NPCs were separated from parenchymal cells by low-speed centrifugation. Hepatic macrophages were separated from other NPCs by their ability to adhere to cell culture plastics within a short time span. The cell morphology and viability of these isolated hepatic cells were checked microscopically. LMs in suspension were identified by their characteristic rounded cell morphology with a prominent nucleus. In contrast, adherent hepatic macrophages show an inhomogeneous and mostly rounded but also partly spindle-shaped morphology (Figure 5). As a result, an average of 1.32 × 10^6^ (±0.95 × 10^6^, SD) viable hepatic macrophages were obtained per gram of liver tissue. In summary, the cell yield and LM morphology vary widely depending on donor pathology and surgical procedures.

### 3.4. Initial Characterization of Liver Macrophages Revealed Anamnesis-Dependent Metabolic and Inflammatory Activation

Freshly isolated and adherent macrophages from patients with varying liver diseases (Table 5) were examined for their initial cell activity and inflammatory states. The initial characterization was performed directly after the isolation procedure and included the evaluation of the cell activity that is also related to cell viability by using the XTT assay. Additionally, the intracellular formation of ROIs as mediators in the NF-κB signaling pathway was measured by using the DCF assay and provides an indication of the functional state of LMs.

**Figure 6:**
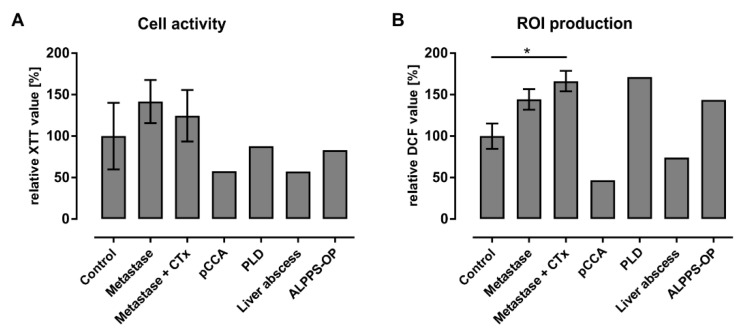
Initial cell activity and inflammatory state of freshly isolated human hepatic macrophages. Anamnesis-dependent analyses of (**A**) cell activity and (**B**) ROI production of human hepatic macrophages. For the initial characterization the (**A**) XTT and (**B**) DCF assays were performed immediately after adherence of freshly isolated liver macrophages. Associated donors are listed in Table 5. Values are normalized to the donors serving as control and data show means ± SD. N: shown inTable 5. *: *p* ≤ 0.05. (ALPPS-OP: “associating liver partition and portal vein ligation for staged hepatectomy” operation, CTx: chemotherapy, PLD: polycystic liver disease, pCCA: perihilar cholangiocarcinoma, ROI: reactive oxygen intermediates).

The investigation of the cell activity of freshly isolated LMs shows distinct donor-dependent variations in all examined diseases (Figure 6A). Isolated hepatic macrophages from donors with liver metastases show slightly increased cell activities compared to the control group. In contrast, cell activities from the patient with pCCA as well as from the donor with liver abscess decreased to approximately 57% in relation to the cells from the control tissues.

With regard to intracellular ROI production, LM cultures from control tissues generally showed consistently lower DCF values than patients with malignant diseases (Figure 6B). Here, the ROI levels in macrophages from patients with liver metastases were nearly 1.4 times higher than those of the control donors. One significant increase in ROI production compared to the control group existed for the group with liver metastases and chemotherapy. Comparing the samples from liver tumor patients with and without chemotherapy, the ROI formation in donors with chemotherapy was only slightly above those in patients without chemotherapy. In contrast, the patient with a Klatskin tumor and the patient with a liver abscess showed a notable decrease in ROI levels to approximately 46% and 74% compared to the control group, respectively.

In summary, the initial characterization of LMs revealed that macrophage activation and inflammatory reactions showed the same tendencies to increase or decrease compared to a control group. Notably, patients with liver metastases showed clearly increased cell and inflammatory activities. In contrast, two donors with pCCA and liver abscess showed reduced metabolic and inflammatory activities.

### 3.5. Characterization of the Adherent NPCs Fraction Showed Various Macrophage Subpopulations

**Figure 7:**
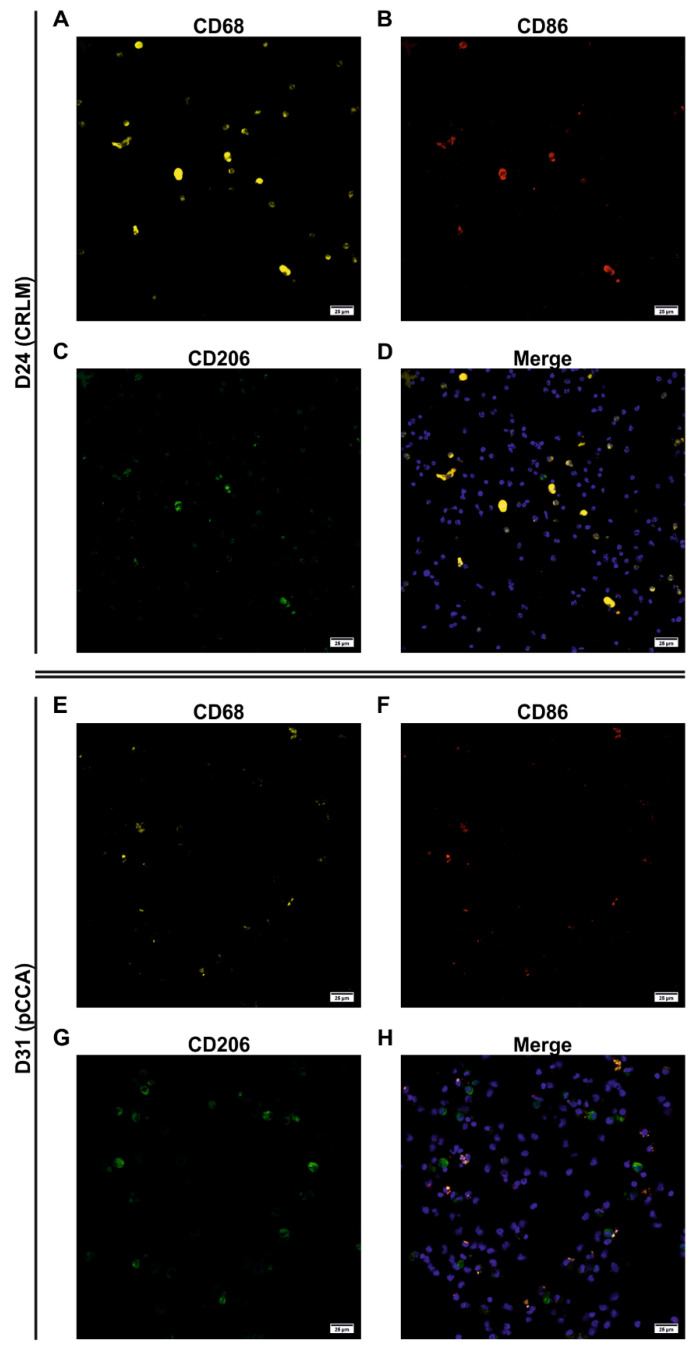
Qualitative evaluation of individual macrophage subpopulations from D24 and D31 (detailed donor data: Table 2). Confocal laser scanning microscopy of adherent and fluorescence-stained human hepatic macrophages was used to assess individual subpopulations. The cells from D24 with CRLM and D31 with pCCA were investigated regarding the marker (**A**,**E**) CD68 for general macrophages (yellow), (**B**,**F**) CD86 for pro-inflammatory (red), and (**C**,**G**) CD206 for anti-inflammatory macrophages (green). (**D**,**H**) The merged images show all markers simultaneously as well as the cell nuclei stained with Hoechst 33342 (blue). Images were taken using a laser scanning microscope (LSM 700, Carl Zeiss). (CRLM: colorectal liver metastasis, pCCA: perihilar cholangiocarcinoma).

The ability to adhere in a short time period is used to separate LMs from other hepatic NPCs and is a standard procedure in the isolation of KCs [22]. Therefore, we investigated this fraction for the presence of inflammation-related subpopulations using immunofluorescent staining and microscopy. Adherent NPCs from tissue samples of four different patients with varying liver pathologies (Table 2) were stained for macrophages. An antibody against CD68 was used to detect macrophages in general, while the expression intensity correlates with the macrophage activation [23]. Additionally, antibodies against CD86 and CD206 were used to identify pro- and anti-inflammatory macrophages, respectively. Cell nuclei were visualized using Hoechst 33342. Cultures from two patient samples showed partly high autofluorescence signals in the controls overlapping with the signals for CD68 and CD86 and/or CD206, limiting the qualitative analysis to three of four patient samples: D24 with CRLM, D31 with pCCA, and D33 with Caroli syndrome (Figure 7, Figures S3 and S4).

The adherent culture from D24 showed CD68+ cells of various sizes. Therefore, larger cells showed a strong signal for CD68 indicating a strong macrophage activation, whereas medium- and small-sized cells showed a diffuse or spotted signal distribution (Figure 7A–D). In contrast, the cultures of D31 (Figure 7E–H) and D33 (Figure S4A–D) showed weaker staining for CD68, which was predominantly diffusely distributed suggesting a weaker macrophage activation. The detection of inflammatory subpopulations revealed that most of the large CD68+ cells from D24 with a strong signal and some medium-sized cells with diffuse signals for CD68 were positive for CD86 at the same time and thus pro-inflammatory (CD68+/CD86+). The staining for CD206 was also slightly diffuse and mostly detected on larger cells, correlating predominantly with CD68+ cells, indicating anti-inflammatory cells. In D33, the majority of diffusely stained CD68+ cells were also CD86+, and only a few cells were CD206+ (Figure S4A–D). In contrast, nearly all CD68+ macrophages from D31 also stained positive for CD206, representing predominantly anti-inflammatory shaped cells (Figure 7E–H).

Additionally, in all cultures, we detected a subpopulation having an unclear inflammatory state (CD68+/CD86+/CD206+) as well as small cells without an inflammatory state (CD68+/CD86− and CD68+/CD206−), and for both CD86 and CD206, we also saw some small, stained cells that were not positive for CD68 (CD68−/CD86+ and CD68−/CD206+).

**Figure 8:**
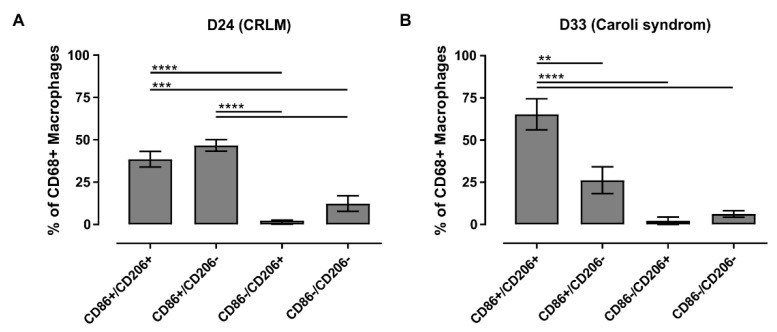
Microscopic quantification of individual subpopulations of liver macrophages. Confocal laser scanning microscopy of adherent and fluorescence-stained human hepatic macrophages was used to analyze individual subpopulations. Staining was quantified for (**A**) patient D24 with CRLM and (**B**) patient D33 with Caroli syndrome and are shown as proportion of CD68+ cells. *n* = 5. Mean ± SD. **: *p* ≤ 0.01, ***: *p* ≤ 0.001, ****: *p* ≤ 0.0001 (CRLM: colorectal liver metastasis).

Moreover, the macrophage cultures from D24 and D33 were evaluable both qualitatively (Figure 7 and Figure S4) and quantitatively (Figure 8). Both cultures accounted for approximately 40% of CD68+ cells in the adherent NPC fraction. The macrophage culture isolated from liver tissue from the patient with CRLM (D24) showed an LM distribution that was dominated by pro-inflammatory macrophages as well as macrophages of unclear inflammatory state (Figure 8A). The macrophage culture isolated from liver tissue from the patient with Caroli syndrome (D33) showed a similar macrophage subpopulation distribution with an even more pronounced fraction of LMs with an unclear inflammatory state (Figure 8B). In both cases, anti-inflammatory macrophages or macrophages without an inflammatory state played a minor or no part in these cultures.

In summary, macrophage activities can lead to autofluorescence signals limiting the microscopic evaluation of adherent LM cultures resulting from NPC fractions. The remaining evaluable cell cultures showed explicit strongly activated pro- (CD68+/CD86+) and weakly activated anti-inflammatory (CD68+/CD206+) macrophages in various yields in accordance with the underlying diseases of the different donors. We also detected hepatic macrophages without an inflammatory state (CD68+/CD86− and CD68+/CD206−) as well as subpopulations with an unclear inflammatory state (CD68+/CD86+/CD206+). Qualitative and quantitative analyses revealed a rather slight pro-inflammatory and clear pro-inflammatory state for D24 and D33, respectively. Qualitative analysis of cell cultures from D31 showed a rather anti-inflammatory state.

### 3.6. FACS Analysis Allowed a More Robust Characterization of Macrophage Subpopulations

As an alternative to fluorescence microscopy, we tested fluorescence-activated cell sorting (FACS) as a method for the initial characterization of LMs. Because adherent cultures can vary in their compositions of macrophage subpopulations from their initial inflammation-specific subpopulations in suspension, we tested both methods in parallel. Therefore, freshly isolated NPCs in suspension were used for FACS analyses using directly labeled antibodies against CD68 characterizing the macrophage population in general, against CD86 as specific for pro-inflammatory macrophages and against CD206 as specific for anti-inflammatory macrophages. Additionally, freshly isolated and adherent hepatic macrophages were analyzed for their cell activity (XTT assay) and inflammatory state (DCF assay) as described above and normalized to the protein content of the cultures (BCA assay). Second, parallel cultures were characterized for their LM subpopulations as described above using immunofluorescence staining and laser scanning microscopy.

In three cases, NPC isolation yielded a sufficient number of cells, allowing this more comprehensive analysis: D18 with echinococcosis, D27 with liver abscess, and D29 with bile duct carcinoma and cholestasis (Table 2).

**Figure 9:**
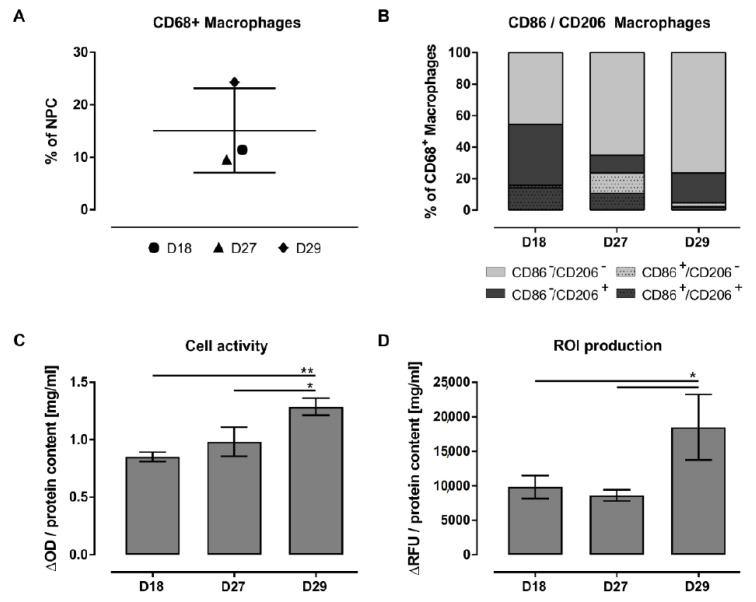
Comprehensive characterization of freshly isolated human hepatic macrophages from distinct liver diseases. Extended investigation of the donors D18 (echinococcosis), D27 (liver abscess) and D29 (bile duct carcinoma and cholestasis; detailed donor data: Table 2). Flow cytometry was used (**A**) to determine the amount of CD68+ macrophages in the NPCs fraction and (**B**) to identify pro- (CD86) and anti-inflammatory (CD206) subtypes of macrophages in the fraction of CD68+ cells. *N* = 3. Additionally, these three donors were considered individually with regard to (**C**) their metabolic activity determined using XTT assay and (**D**) their intracellular ROI production determined using DCF assay. XTT and DCF values are normalized to the protein content. *N* = 3. Mean ± SD. *: *p* ≤ 0.05, **: *p* ≤ 0.01. (NPCs: non-parenchymal cells, ROI: reactive oxygen intermediates).

Flow cytometric analysis of the proportion of macrophages in the isolated NPC cell suspension showed an average of 10.5% (±0.9%) macrophages for liver tissue from a patient with echinococcosis (D18) and from a patient with liver abscess (D27). In comparison to these subjects, a liver tissue sample from a patient with bile duct carcinoma and hepatic cholestasis (D29) showed an increased LM share of approximately 24% and was thus more than two times higher (Figure 9A). Additionally, the FACS data confirmed the presence of the same macrophage subpopulations already detected with immunofluorescent staining in adherent cultures (Figure 9B).

The liver tissue from a patient with echinococcosis (D18), used here as a control, was characterized by macrophages showing an anti-inflammatory state (53%) and by macrophages without an inflammatory state (45%). Additionally, approximately 27% of CD206+ cells and thus 14% of the CD68+ macrophages had an unclear inflammatory state (CD68+/CD86+/CD206+). The proportion of explicit pro-inflammatory LMs was very small at 1%. The most striking change in the other two investigated tissues (D27 and D29) is the increase in the macrophage fraction without an inflammatory state to 65% and 76% and the decrease in the macrophage share with an unclear inflammatory state to 11% and 2%, respectively. In addition, macrophages from the tissue with liver abscess (D27) showed the greatest CD86+ pro-inflammatory cell population of the investigated donors, at approximately 23%. In contrast, the tissue from the patient with bile duct carcinoma and cholestasis (D29) showed a large population of explicit anti-inflammatory cells of approximately 19%.

Characterization of adherent NPCs revealed high autofluorescence signals in all three channels for CD68, CD86, and CD206 in the control cultures of all three investigated donors. However, in the cultures from D18 (Figure S5) and D27 (Figure S6), these autofluorescence signals were partly distinguishable from specific stainings, allowing a qualitative approach. In particular, in the culture from D18, we observed that autofluorescence signals were often spots identified as accumulations within the cells. In addition, the cells showed diffuse but distinct signals for all three markers, whereas almost all cells were positive for CD68 with less contamination of CD68− cells. From this CD68+ fraction, some cells were CD86+, but a large proportion were CD206+, showing mostly anti-inflammatory shaped cells (Figure S5). Thus, the results from the adherent culture correspond with those from the NPCs suspension investigated using FACS analysis. The adherent cells from D27 also showed only a few signals for CD86 but many distinct CD206+ cells, of which some cells were positive for CD86 at the same time (Figure S6). In comparison to the FACS analysis, the pro-inflammatory CD86+ fraction appears to be underrepresented.

Investigations of the isolated, adherent LMs for cell activity confirmed that the pathologies from the diseased donors led to more active macrophages in contrast to the tissue from the controls (Figure 9C). This observation was significant for macrophages from tissue with bile duct carcinoma and cholestasis (D29), which also showed increased inflammatory products by significantly elevated intracellular ROI levels (Figure 9D).

Taken together, the FACS analysis revealed that liver pathologies were characterized by their amount of LMs as well as by their differentiation into inflammatory subpopulations. All donors showed subpopulations with clear pro- and anti-inflammatory states in various proportions depending on their underlying diseases but also cells with an unclear or without an inflammatory state. The evaluable results regarding the inflammatory subpopulations on adherent hepatic macrophages were partly consistent with the FACS outcome showing in both methods mostly anti-inflammatory cells in the control sample from a patient with echinococcosis. In contrast, the pro-inflammatory fraction from a donor with liver abscess looked underrepresented in the adherent culture compared to the cells in suspension. Parallel measurement of the cell activity showed a correlation of increased cell activity with an increased proportion of LMs with an unclear inflammatory state. The fact that the intracellular ROI level was significantly elevated only in the rather anti-inflammatory characterized tissue from a patient with bile duct carcinoma and cholestasis showed that an increased intracellular ROI level is not an explicit pro-inflammatory feature.

## 4. Discussion

KCs are resident liver macrophages that play a central role in hepatic tissue homeostasis. In their quiescent state, they are responsible for blood cell, microbe, and particle clearance. Once activated, they differentiate into pro- and anti-inflammatory subpopulations responsible for elimination of stressed and dead hepatocytes or immune tolerance induction, respectively. In liver pathologies, KCs receive support from MDMs to eliminate damaged tissue and to initiate regenerative processes. The investigation of these processes in humans requires in vitro liver models with the participation of isolated primary human LMs. Therefore, the aim of this study was to gain further insight into the participation of hepatic macrophages in different liver pathologies as well as into their characteristics once isolated from human liver tissues.

### 4.1. Selectivity of Specific Antigens for KC Characterization

We used specific antibodies for staining LMs in general (CD68) and for staining activated macrophages differentiating into pro- (CD80 and CD86) and anti-inflammatory (CD163 and CD206) subpopulations. Traditionally, CD68 is the gold standard for macrophage identification, as this protein is highly expressed in cells of the mononuclear phagocyte lineage, including monocytes, macrophages and dendritic cells (DCs) [24]. Monocytes and macrophages can express CD68 depending on their activation and environmental stimuli [25]. Once activated, they can bind to the vessel walls and accumulate at the sites of injury or inflammation [3,7]. Therefore, their detection as macrophages cannot be excluded. In human DCs, the anti-CD68 reactivity is located in a discrete juxtanuclear spot in contrast to macrophages which have CD68-reactivity throughout the cytoplasm expressed on lysosomes [26]. Several studies have shown a significant upregulation of CD68 on macrophages in response to various stimuli [27]. Thus far, it is not possible to distinguish between KCs and recruited macrophages in humans [7]. Therefore, CD68 is not specific for KCs but for macrophages in general when the detection method allows their separation from DCs by taking into account the location of the expression. Additionally, the expression level allows conclusions on macrophage activation.

In this study, CD80 and CD86 were used as markers for pro-inflammatory activated macrophages. These two B7 family members are co-stimulatory molecules that regulate T cell responses [28,29] and have limited expression on professional antigen-presenting cells (APCs) such as macrophages, DCs and activated B cells [30]. In contrast, no CD80 or CD86 immunoreactivity could be observed on hepatocytes, bile ducts or hepatic stellate cells [31]. Leifeld and co-workers demonstrated that these markers detect macrophages in human liver tissues and that only a few KCs and no liver sinusoidal endothelial cells express CD80 and CD86 in “healthy” individuals, in contrast to patients with acute or chronic inflammatory liver diseases [31]. The scavenger receptors CD163 and CD206 were used as markers for anti-inflammatory activated resident LMs, which are upregulated in response to inflammation and thus allow the detection of the activation of macrophages, including KCs [32]. CD163 is exclusively expressed by monocytes and macrophages [33], while CD206 is primarily expressed by selected populations of macrophages, including KCs and DCs, and by lymphatic and hepatic endothelial cells [32]. Both endocytic receptors are associated with phagocytosis [23] and are continuously recycled between the cell surface and early endosomal compartments so that in homeostasis, only a small amount of these markers is present at the cell surface [32].

Taken together, it is well known that LMs in the healthy state and in disease are heterogeneous cell populations, and no exclusive markers exist for their discrimination [3,11]. Nevertheless, the chosen markers used in this study are at least highly specific for hepatic phagocytes and detect macrophages and their progenitors as well as other APCs, including DCs, and the hepatic endothelium.

### 4.2. The majority of Investigated Liver Pathologies Are Characterized by Anti-Inflammatory Macrophages

For the characterization of different liver pathologies, we investigated 15 different liver tissue samples from patients with varying underlying diseases. We stained for LMs in general (CD68) as well as their pro- (CD80) and anti-inflammatory (CD163) subpopulations and used a subsequent automated imaging analysis for marker quantification. Direct comparison of stained markers of equal sections from the tissue slides revealed a good correlation of the CD68+ macrophages with the expression of CD80 and CD163. However, there are multiple examples of high numbers of CD80+ and CD163+ cells without parallel expression of CD68, suggesting the presence of other immune cells carrying these markers. Low numbers of CD68+ cells were found in the liver tissue sections of the control group indicating low macrophage activity in association with benign diseases; additionally, in D10 with diagnosed HCC and cirrhosis, the findings also indicated low macrophage activity in chronic diseases. In contrast, an increased number of CD68+ macrophages was observed in patients with Klatskin tumors (D06-D08) as well as in one patient diagnosed with HCC (D09), indicating high macrophage activity. The latter donor with an increased number of hepatic macrophages also had an accompanying high number of CD163+ cells. This high number of anti-inflammatory macrophages indicates chronic inflammation as well. This result is in line with our histologic evaluation showing a multitude of fibrotic tissue changes consistent with cirrhosis. It is known that advanced fibrosis and cirrhosis based on failed and ongoing regenerative events are caused by anti-inflammatory and pro-fibrotic M2 macrophages [34,35]. Therefore, a high number of CD163+ cells can correlate with fibrosis and cirrhosis, as seen in D09, but their number can also be increased, as seen in D15, without any signs of pathology.

In the hepatic tissue sections examined here, CD163+ macrophages were distributed quite homogeneously in the liver lobules with only a slight tendency to form clusters. A main location of anti-inflammatory cells in the periportal area, as reported by Guillot and Tacke [3], could not be observed. In contrast, we observed pro-inflammatory actions by CD80+ cells in regionally limited areas. This lobule-specific pattern was observable either pericentral (D04), periportal (D05), or both (D13), but was also randomly distributed over the whole tissue section (D08). It was demonstrated in a mouse model that a pericentral accumulation of LMs can be a specific feature of CCA [36], similar to our observation of the pro-inflammatory macrophage distribution in a patient with iCCA (D04). The periportal concentration and ring-like appearance of the CD80+ cells on the lobule borders found in the tissue of D05 with diagnosed HCC could indicate a defined area of injury formed by MDMs as described elsewhere [7]. Regarding fibrotic livers, patient D08 with pCCA showed a tendency for CD80+ macrophages to accumulate in the scars, with very few of these cells in the parenchymal tissue, in line with another study in which pro-inflammatory antigens were also predominantly found in cells of the septa [10]. In contrast, D07 showed no accumulation of pro-inflammatory LMs in the scars. Instead, the CD80+ cells showed a tendency to concentrate around the lipid droplets in the parenchymal areas in both patients D07 and D08. It is known that liver regeneration of larger tissue parts, e.g., after liver surgical resection, induces short-term steatosis [37] as a consequence of regeneration-related ER stress [38]. Therefore, we conclude that these lipid-rich spots show areas of tissue damage with early events of regeneration. In contrast, a general increase in hepatic lipid accumulation, as described for patients D04 and D14, showed no striking changes in macrophage populations, even if severe steatosis or NAFLD was observable (D03 and D10).

Taken together, our data show that the majority of our investigated liver tissues are characterized by anti-inflammatory cells. These CD163+ macrophages show a homogeneous distribution, and increased cell numbers mostly correlate with chronic liver injuries. In contrast, pro-inflammatory LMs appear as a temporary and locally restricted reaction.

### 4.3. Patients Diagnosed with Klatskin Tumors Showed Acute to Chronic Progression of Inflammation

Our analysis revealed a striking correlation of the LM number with fibrosis progression in donors with pCCA (D06-D08). Due to its prominent position, a pCCA seals the central bile duct in a short time period, leading to acute cholestasis in a large region of the liver. Therefore, depending on the time point, cholestasis, which is reversible at first, can lead to increased liver stiffness and subsequently to fibrosis and cirrhosis [39]. This is in line with Ju and Tacke, who emphasize that a large number of MDMs are a hallmark feature of acute and chronic liver injury in mice and humans [7]. In our study, CD68+ macrophages tended to accumulate with fibrosis progression (D06-D08). From experiments in rodents, it is known that inflammatory monocytes recruited into the injured liver promote the progression of liver fibrosis. During chronic liver damage, MDMs differentiate preferentially into inducible NO synthase-producing macrophages exerting pro-inflammatory and pro-fibrogenic actions [40]. These results are in line with our observations showing a very high number of LMs accompanied by a high number of CD80+ cells and low fibrosis in D08, indicating acute liver damage. In contrast, high numbers of LMs were accompanied by a low number of CD80+ cells and moderate fibrosis in D07 and were accompanied by an increased number of CD163+ cells and advanced fibrosis in D06, indicating chronic liver damage. This is in accordance with the results from Beljaars et al., who also showed that pro- and anti-inflammatory macrophages are present side by side in fibrotic lesions, while the persistence of pro-inflammatory LMs seems to cause a regression of fibrosis in both mouse and human liver tissues [8]. Similar observations were also observed in a mouse model of biliary obstruction where hepatic macrophages suppressed cholestatic liver injury by cytokine-dependent mechanisms, including the production of liver regeneration-associated IL-6 [41]. We conclude that short-term cholestasis leads to a pro-inflammatory reaction mediated by CD80+ cells, while chronic tissue damage is associated with a shift to an increasing number of MDMs with a dominant anti-inflammatory CD163+ cell fraction.

### 4.4. Isolation of Primary Human Non-Parenchymal Liver Cells Resulted in a Macrophage-Rich Fraction Showing Donor-Related Activation and Reactivity

The adherent macrophage-rich NPC fraction was characterized with respect to macrophage activation by measuring cellular activity using the XTT assay and the intracellular ROI level using the DCF assay. Previous studies on isolated LMs have already shown the relevance of intracellular ROIs as signaling molecules in the NF-κB pathway of macrophages [42,43], indicating pro-inflammatory reactions. Kegel et al. demonstrated by performing ROI measurements that hepatic macrophages are already partially activated after isolation (initial LM activation). Additionally, the study concluded that there is a correlation between donor anamnesis and LM activation that is proportional to the time span since induction and to the intensity of the tissue damage, respectively [21]. The adherent NPC fractions from the donors with chemotherapy showed a tendency toward higher cell activity accompanied by significantly increased ROI production compared to the cells from control liver tissues. Recent findings describe long-lasting and partly persisting liver sinusoidal vessel changes after chemotherapy of CRLM termed hepatic sinusoidal obstruction syndrome [44]. It is thought that such changes also affect the sinusoidal-associated KCs. However, our investigation of tissues did not show an increase in pro-inflammatory macrophages in D02 or D03 with CRLM and treatment with chemotherapy. In contrast, clearly decreased cellular activity and intracellular ROI values were observed in a donor with pCCA and cholestasis (D25), indicating low macrophage activation in patients with a chronic disease. Kegel et al. demonstrated a very low cell activity and an anti-inflammatory response for isolated LMs of a donor with Klatskin tumor after incubation with supernatants of stressed PHH [21]. Therefore, we conclude that the anti-inflammatory cell fraction observed in tissue sections from pCCA consists of LMs characterized by low cell activity and decreased NF-κB activation.

Further characterization of adherent NPCs cultures from patients with different liver pathologies was performed by immunofluorescent staining for hepatic macrophages. In general, most of the cultures showed strong autofluorescence signals which did not allow any qualitative or quantitative evaluation. Several studies have observed autofluorescence signals and have cited macrophages and neutrophils as the reason. In general, immune cells tend to be more autofluorescent than other cell types, which could be observed in particular in large macrophages due to their high content of flavoprotein-associated granules [45]. Therefore, the presence of autofluorescent cell types correlates with increased phagocytic activity, whereas phagocytosis was, in turn, identified as a trigger to switch from pro-inflammatory MDMs to an anti-inflammatory and restorative phenotype in mice [7]. These observations are in line with our data for D31 showing moderate signs of autofluorescence, which was characterized by quite large macrophages and a primarily anti-inflammatory cell population.

In the case of two donors, the examination of quantitatively evaluable adherent cultures yielded a proportion of approximately 40% CD68+ cells, indicating contamination with other activated cells of the immune system. Qualitative analysis of the adherent NPCs cultures showed CD68+ macrophages with clear pro-inflammatory characteristics. The additional presence of many CD68- cells that stained positive for CD86 in the culture of D24 and D33 suggests the presence of further pro-inflammatory APCs, such as activated B lymphocytes or monocytes, which express the costimulatory protein. In healthy livers, KCs are described as macrophages with a predominantly anti-inflammatory character, while activated and infiltrating MDMs are described as macrophages with a predominantly pro-inflammatory character [25]. Additionally, we observed LMs with an unclear (CD86+/CD206+) and without (CD86−/CD206−) an inflammatory state. CD86+/CD206+ cells were described as an immunoregulatory subpopulation of M2 macrophages, namely, M2b [46,47]. This subpopulation is mainly induced by immune complexes and stimulation with Fc receptors and exerts strong immune-regulating and anti-inflammatory effects but possesses both protective and pathogenic roles in various diseases [48]. The observation of a CD86−/CD206− macrophage population without inflammatory characteristics suggests the presence of CD68+ monocytes or quiescent macrophages probably freshly derived from monocytes or macrophages with marker expression below our detection limit [49].

Quantitative analysis of the microscopic images of the CD68+ macrophages from D24 revealed mainly pro-inflammatory CD86+ and M2b macrophages. The large proportion of pro-inflammatory LMs correlates with a diagnosed acute florid inflammation of the liver tissue. The fact that D24 with CRLM was additionally treated with chemotherapy supports the suggestion that chemotherapeutic treatments of secondary tumors can lead to a long-lasting activation of macrophages, probably with M2b participation. The M2b population was also found in an even higher quantity in the patient with Caroli syndrome (D33), representing in this case the majority of the macrophage subpopulations. This is in line with the underlying disease, which is characterized by a chronic course with inflammation of the bile ducts and liver fibrosis as well as ongoing regenerative processes such as epithelial hyper- and metaplasia. Macrophages with a pro-inflammatory state can be related to acute inflammation, probably due to cholestasis-related bacterial invasion [50].

In contrast, D31 with a pCCA showed an LM population with a primarily anti-inflammatory character, as already observed in liver tissue sections with chronic inflammation. Additionally, D31 showed CD68-/CD206+ cells, suggesting the presence of DCa and underlining the anti-inflammatory character of the adherent NPC culture.

Taken together, our characterization of the adherent NPC fractions from liver tissue samples showed a CD68+ macrophage yield of approximately 40%. Contaminating cells showed immune cell characteristics that are in line with the inflammatory character of the LMs. Pro-inflammatory macrophage cultures are characterized by high cellular activity and an increased intracellular ROI level, indicating NF-kB pathway activation. Additionally, these cultures show macrophage subpopulations with an unclear inflammatory character representing immune modulatory macrophages of the M2b type. Anti-inflammatory macrophage cultures are characterized by low cellular activity and decreased ROI levels. Additionally, these cultures show large macrophages with a strong autofluorescence, indicating augmented phagocytic activity and anti-inflammatory characteristics. Finally, the adherent NPC cultures contained LMs without inflammatory characteristics, probably representing freshly activated monocytes.

### 4.5. Isolated Primary Hepatic Macrophages Are a Heterogeneous Cell Fraction with Predominant Anti-Inflammatory Characteristics

For three donors, simultaneous in vitro analyses using flow cytometry, immunofluorescent staining, and XTT and DCF assays were possible to comprehensively characterize their macrophages. The characterization of NPCs in suspension using flow cytometry demonstrated that the proportion of CD68+ cells in the control liver tissues was on average 10%, in contrast to a more than two times higher proportion of CD68+ cells in the case of one donor with bile duct carcinoma and cholestasis. KCs accounted for 35% of the hepatic NPC fraction [51]. However, the fact that CD68 expression can be low in homeostatic LMs may result in a high number of undetected cells when using FACS analysis.

The patient with echinococcosis (D18) representing the control sample showed an anti-inflammatory phenotype with predominantly CD206+ cells in the FACS analysis and many large autofluorescent cells in the adherent culture. This macrophage pattern corresponds to a tolerogenic initial state of the control liver tissues [12]. Low XTT and intracellular ROI production of the macrophages confirm an anti-inflammatory character of the cell culture. Therefore, we conclude that in our control livers, the macrophage population shows a distinct phagocytosis-active KC population with anti-inflammatory characteristics. The proportion of LMs without an inflammatory state suggests an activated monocyte population. The fact that they appear in adherent cultures only in low numbers indicates limited attachment qualities, confirming that they are monocytic cells. As they do not play a critical role in healthy livers, monocytes are either activated by surgery or the isolation process or contaminating blood monocytes.

A patient with a liver abscess (D27) showed a larger number of CD68+ cells without an inflammatory state compared to the donor with echinococcosis (D18) but also the largest proportion of pro-inflammatory cells in the FACS analysis. Both macrophage subpopulations were clearly underrepresented in the immunofluorescent staining of the adherent culture. There, the detection of anti-inflammatory CD206+ macrophages, autofluorescent cells indicating phagocytic macrophages, and a CD86+/CD206+ subpopulation representing the regulatory M2b macrophage type accounts for an anti-inflammatory state. In addition, low macrophage activity and intracellular ROI production confirm the anti-inflammatory character of the adherent culture. A pro-inflammatory macrophage pattern would fit with the underlying disease, as an abscess is accompanied by an inflammatory process caused by the occurrence of bacterial infections [14]. Therefore, FACS analysis showed a macrophage pattern that fits the underlying disease of the patient. We conclude that the adherent cultures show only a selection of the LM population, probably shifted by the cell isolation process or selection methods. In particular, CD86-/CD206- and pro-inflammatory CD86+ cells can be populations that are underrepresented in primary macrophage cultures.

The patient with bile duct carcinoma and cholestasis (D29) showed the largest number of CD68+ cells without an inflammatory state. The remaining inflammatory cells of D29 showed a distinct anti-inflammatory phenotype, which is in line with the chronic progression of the underlying disease. Additionally, high macrophage activation in D29 could be linked to inflammatory reactions in the context of previously performed portal vein embolization. The high number of LMs shows similarities to the results seen in the tissues from the donor group with pCCA. Increased cell activity and ROI production are signs of pro-inflammatory reactions fitting with the acute to chronic progression, as seen in the early stages of tissue damage due to pCCA. Therefore, we conclude that the surplus of LMs in these donors is freshly activated monocytes, which are at least in the early stages characterized by the lack of an inflammatory state.

**Figure 10:**
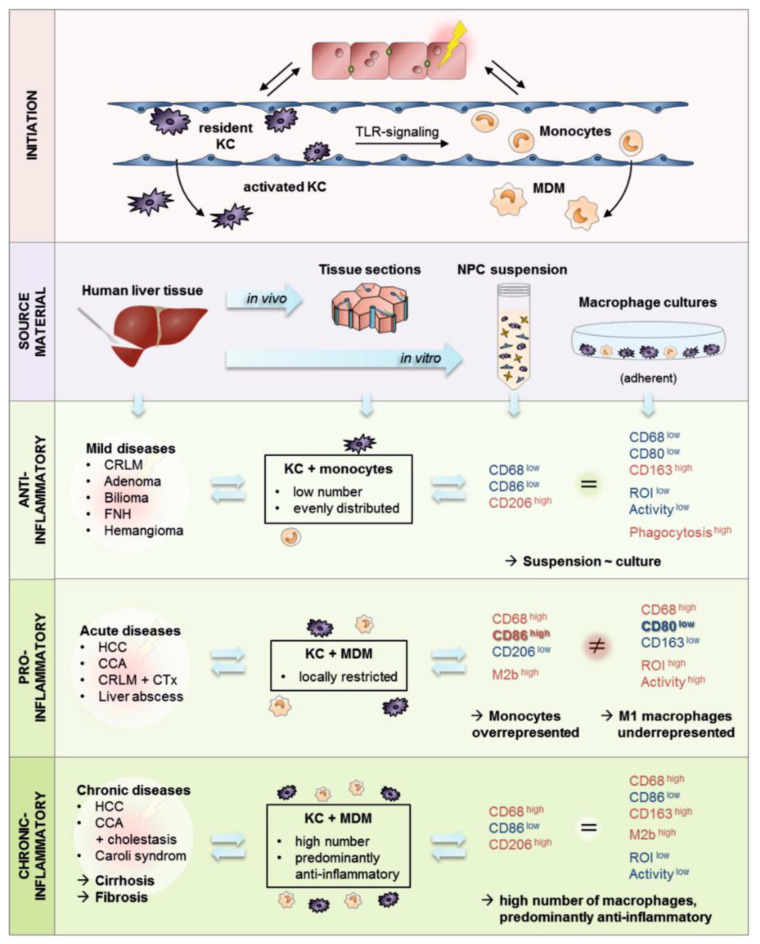
Summary of the results. For this study, we investigated human LMs in tissue sections as well as isolated from human liver tissues which were characterized both in suspension and in adherent cultures. Our data revealed that the inflammatory state of primary human LMs corresponds to patients and disease characteristics and that it is possible to maintain the macrophage pattern in suspension as well as in adherent cultures with just a few limitations. (CCA: cholangiocarcinoma, CRLM: colorectal liver metastasis, FNH: focal nodular hyperplasia; HCC: hepatocellular carcinoma, LMs: liver macrophages, MDMs: monocyte-derived macrophages, NPCs: non-parenchymal cells, TLR: toll-like receptor).

In summary, our data revealed differences in the macrophage population when analyzed as a cell suspension directly after isolation by FACS or as an adherent culture using immunofluorescent staining (Figure 10). The FACS analysis was dominated by CD68+ cells without inflammatory characteristics that were missing in the adherent cultures. The data suggest that these cells are activated monocytes expressing CD68 that accumulate in the liver. However, only a very small fraction showed an adhesion ability and were therefore relevant to the LM population. Thus, FACS analysis is affected by a high number of monocytes. In contrast, pro-inflammatory macrophages can be lost or die during the cell isolation and separation steps.

### 4.6. Limitations of the LM Characterization

Our study is limited by the numbers of donors we investigated. Additionally, the usage of primary cells is accompanied by high individual variances depending on various factors such as age, sex, underlying disease, lifestyle, and medication. Within these limitations, we decided for an analysis using various samples and methods to confirm our results with different read-outs, leading to a consistent analysis and revealing a plausible picture.

Even if our chosen markers are highly specific for macrophages, they cannot differentiate between KCs and MDMs. However, increased numbers of LMs, as observed in fibrotic tissues, suggest a high participation of MDMs in the inflammatory process. Additionally, the expression level of the investigated markers can vary, resulting in an underestimation of macrophages in general as well as of specific macrophage subpopulations. Moreover, the occurrence of regulatory macrophages of the M2b type as well as of monocytes requires additional markers for their identification and characterization [48,52].

## 5. Conclusions

Our study clearly showed that the macrophage subpopulations identified using FACS analysis showed high comparability to the patient and disease characteristics. In most cases, this macrophage pattern can be transferred to adherent cultures. These data confirm that it is possible to maintain the inflammatory state in adherent cultures and that these correspond to the patients and disease characteristics (Figure 10). The high number of contaminating cells can be useful for complex liver models while displaying a comprehensive inflammatory situation, but it can also be a drawback in simple models used for investigations of mechanistic processes. Therefore, if a higher purity of the macrophage fraction or more defined subpopulations is needed, further clean-up procedures in the isolation process, such as MACS or cell sorting using flow cytometry, are necessary. MDMs were reported to have a short half-life and therefore can be a critical component in liver models. Thus, KCs are considered the more stable macrophage type and are therefore essential for long-term cultures. To specifically isolate KCs, the usage of tissue samples rich in KCs and poor in MDMs is required but represents a very limiting factor. The rarity of such “healthy” tissue samples is a major drawback for research on KCs. Therefore, we recommend the isolation of hepatic cells from patients with specific liver diseases depending on the research topic, as the phenotype of the cells is related to them. Additionally, the differentiation of KC-like cells from monocytes and monocytic cell lines could be an alternative and should be considered for future investigations.

## Figures and Tables

**Table 1:** Summarized donor data for liver tissue samples used for immunohistochemical staining. Donor data are summarized based on the underlying disease. In addition, age; sex as well as histopathological findings for steatosis, fibrosis and inflammation of the donors are indicated. For details on clinical data, see Table S1 in the Supplementary Materials.

Summary	Donor ID	Age, Sex	Disease	Steatosis	Portal Fibrosis	Portal Inflammation
**Primary liver tumors** (*N* = 7)	**D05**	77, f ^1^	HCC ^2^, low cholestasis	5%	moderate	mild
	**D09**	59, m ^3^	HCC, cirrhosis	5%	slight to moderate	mild to moderate
	**D10**	79, f	HCC, Child A cirrhosis	60%, NAFLD ^4^/AFLD ^5^	cirrhosis	moderate
	**D04**	46, f	iCCA ^6^, low cholestasis	15%	moderate	moderate, mild florid
	**D06**	77, f	pCCA ^7^, moderate cholestasis	5%	partly distinct, also portoportal	mild, also periportal
	**D07**	65, f	pCCA, moderate cholangitis and cholestasis	5%	slight	mild
	**D08**	75, m	pCCA, moderate cholestasis	5%	moderate	mild to moderate, also periportal
**Secondary liver tumors** (*N* = 3)	**D02**	74, f	CRLM ^8^ + CTx ^9^	5%	-	mild
	**D03**	50, m	CRLM + CTx	NAFLD/AFLD	slight	mild
	**D01** (control)	38, f	CRLM	-	low	mild to moderate
**Benign liver tumors** (*N* = 5)	**D11** (control)	69, m	Bilioma (infected)	-	highly chronic, partly granulating	moderate florid
	**D12** (control)	24, f	Adenoma	-	slight	mild
	**D13** (control)	41, m	FNH ^10^	5%	slight	mild
	**D14** (control)	26, f	Adenoma	75%	slight to moderate	mild
	**D15** (control)	55, f	Hemangioma	5%	slight	mild

^1^ f: female, ^2^ HCC: hepatocellular carcinoma, ^3^ m: male, ^4^ NAFLD: non-alcoholic fatty liver disease, ^5^ AFLD: alcoholic fatty liver disease, ^6^ iCCA: intrahepatic cholangiocarcinoma, ^7^ pCCA: perihilar cholangiocarcinoma, ^8^ CRLM: colorectal liver metastasis, ^9^ CTx: chemotherapy, ^10^ FNH: focal nodular hyperplasia.

**Table 2:** Donor data for liver tissue samples used for the isolation of primary human liver cells. Summary of donors including age, sex, as well as medical history.

Donor ID	Age, Sex	Disease	Medical History
**D16**	59, f ^1^	Angiomyolipoma	-
**D17**	28, f	FNH ^2^	IgA-nephropathy
**D18**	28, m ^3^	Echinococcosis	-
**D19**	45, m	CRLM ^4^	ulcerative colitis, s/p ^5^ colectomy
**D20**	39, m	CRLM	recurrence
**D21**	64, m	CRLM	CTx ^6^
**D22**	61, f	Metastasized cervix carcinoma	CTx, steatohepatitis
**D23**	46, f	CRLM	CTx, steatohepatitis
**D24**	78, m	CRLM	recurrence, CTx
**D25**	72, m	pCCA ^7^	s/p prostate cancer
**D26**	42, f	Hereditary polycystic liver and kidney disease	immunosuppressive therapy
**D27**	47, m	Liver abscess	infectious genesis, s/p adenocarcinoma
**D28**	49, m	CRLM	in-situ split liver resection, avital tissue
**D29**	66, m	Bile duct carcinoma	cholestasis, s/p PVE
**D30**	74, m	pCCA	cholangitis
**D31**	69, f	pCCA	s/p PVE ^8^, cholangitis
**D32**	77, m	HCC ^9^	urothelial carcinoma, obesity, intravesical CTx
**D33**	47, f	Caroli disease	cholangitis, s/p acute hepatitis, s/p intraabdominal abscess, obesity

^1^ f: female, ^2^ FNH: focal nodular hyperplasia, ^3^ m: male, ^4^ CRLM: colorectal liver metastasis, ^5^ s/p: status post, ^6^ CTx: chemotherapy, ^7^ pCCA: perihilar cholangiocarcinoma, ^8^ PVE: portal vein embolization, ^9^ HCC: hepatocellular carcinoma.

**Table 3:** Unconjugated primary as well as secondary antibodies for immunohistochemical stainings of human liver tissue sections. Summary of antibodies including antigen, conjugate, macrophage type, clone, isotype and host species, dilution, as well as incubation time and temperature.

**Primary Antibodies ^1^**						
**Antigen**	**Macrophage Type**	**Clone**	**Isotype, Host Species**	**Dilution**	**Incubation Time**	**Incubation Temperature**
**CD68**	Miscellaneous	KP1	IgG1, mouse	1:150	overnight	4 °C
**CD80**	M1	EPR1157(2)	IgG, rabbit	1:1000	overnight	4 °C
**CD163**	M2	OTI2G12	IgG1, mouse	1:150	overnight	4 °C
**Secondary Antibodies ^2^**						
**Antigen**	**Conjugate**	**Clone**	**Host Species**	**Dilution**	**Incubation Time**	**Incubation Temperature**
**Mouse-IgG**	Peroxidase	polyclonal	rabbit	1:200	2 h	RT ^3^
**Rabbit-IgG**	Peroxidase	polyclonal	goat	1:200	2 h	RT

^1^ Purchased from Abcam (Cambridge, United Kingdom) and diluted in TBS supplemented with 1% BSA and 0.03% Triton X-100, ^2^ Purchased from Sigma and diluted in TBST, ^3^ RT: room temperature.

**Table 4:** Antibodies for immunofluorescence staining for flow cytometry as well as of adherent cells. Summary of antibodies including antigen, fluorophore, macrophage type and localization, clone, isotype and host species, dilution as well as incubation time, and temperature.

Conjugated Antibodies ^1^							
Antigen	Fluorophore	Macrophage Type, Localization	Clone	Isotype	Dilution	Incubation Time	Incubation Temperature
**CD68**	PE	Miscellaneous, intracellular	REA886	IgG1 (human recombinant)	1:50	10 min	RT ^2^
**CD86**	APC-Vio700	M1, extracellular	REA968	IgG1 (human recombinant)	1:50	10 min	4 °C
**CD86**	PerCP-Vio700	M1, extracellular	FM95	IgG1κ (mouse)	1:11	10 min	4 °C
**CD206**	FITC	M2, extracellular	DCN228	IgG1κ (mouse)	1:11	10 min	4 °C

^1^ Purchased from Miltenyi (Bergisch Gladbach, Germany), ^2^ RT: room temperature.

**Table 5:** Grouping of liver tissue samples used for initial characterization of isolated human liver macrophages. Donor data are summarized based on the underlying disease. In addition, age, sex, as well as medical history are indicated.

Summary	Donor ID	Age, Sex	Disease	Medical History
**Control** (*N* = 3)	**D16**	59, f ^1^	Angiomyolipoma	-
	**D17**	28, f	FNH ^2^	IgA-nephropathy
	**D18**	28, m ^3^	Echinococcosis	-
**Metastase** (*N* = 2)	**D19**	45, m	CRLM ^4^	ulcerative colitis, s/p ^5^ colectomy
	**D20**	39, m	CRLM	recurrence
**Metastase + CTx**^6^ (*N* = 4)	**D21**	64, m	CRLM	-
	**D22**	61, f	Metastasized cervix carcinoma	steatohepatitis
	**D23**	46, f	CRLM	steatohepatitis
	**D24**	78, m	CRLM	recurrence
**pCCA**^7^ (*N* = 1)	**D25**	72, m	Klatskin tumor	s/p prostate cancer
**PLD**^8^ (*N* = 1)	**D26**	42, f	Hereditary polycystic liver and kidney disease	immunosuppressive therapy
**Liver abscess** (*N* = 1)	**D27**	47, m	Liver abscess	infectious genesis, s/p adenocarcinoma
**ALPPS-OP**^9^ (*N* = 1)	**D28**	49, m	CRLM	in-situ split liver resection, avital tissue

^1^ f: female, ^2^ FNH: focal nodular hyperplasia, ^3^ m: male, ^4^ CRLM: colorectal liver metastasis, ^5^ s/p: status post, ^6^ CTx: chemotherapy, ^7^ pCCA: perihilar cholangiocarcinoma, ^8^ PLD: polycystic liver disease, ^9^ ALPPS-OP: “associating liver partition and portal vein ligation for staged hepatectomy” operation.

## Data Availability

The data presented in this study are available on request from the corresponding author. The data are not publicly available due to data protection regulations.

## Supplementary Materials

The following are available online at https://www.mdpi.com/article/10.3390/biomedicines9040406/s1, Table S1: Detailed donor data for liver tissue samples used for immunohistochemical stainings of the targets CD68, CD86 and CD206, Figure S1: Gating strategy for Flow cytometric analysis, Figure S2: Determination of LM subpopulations in human liver tissue sections by analyzing parallel immunohistochemical stainings, Figure S3: Autofluorescence controls for the qualitative evaluation of individual LM subpopulations from D24 and D31, Figure S4: Qualitative evaluation of individual LM subpopulations from D33, Figure S5: Qualitative evaluation of individual LM subpopulations from D18, Figure S6: Qualitative evaluation of individual LM subpopulations from D27. The whole tissue scans of all donors and immunohistochemical stainings are accessible by using the platform ‘LiSyM SEEK’ (https://seek.lisym.org/investigations/25 (accessed on 8 April 2021)).

## Abbrevations

ALPPS	“associating liver partition and portal vein ligation for staged hepatectomy” operation
APCs	Antigen-presenting cells
BCA	Bicinchoninic acid
BSA	Bovine serum albumin
CC	Cholangiocytes
CRLM	Colorectal liver metastasis
CRP	C-reactive protein
CSV	Comma Separated Values
CTx	Chemotherapy
CZI	Carl Zeiss Image
DAB	3,3’-Diaminobenzidine
DCs	Dendritic cells
DCF	2,7–dichlorofluorescein
DCF-DA	2,7–dichlorofluorescein diacetate
DILI	Drug induced liver injuries
ECM	Extracellular matrix
EDTA	Ethylenediaminetetraacetic acid
ER	Endoplasmic reticulum
FACS	Fluorescence activated cell sorting
FMO	Fluorescence Minus One
FNH	Focal nodular hyperplasia
HBV	Hepatitis B viruses
HCC	Hepatocellular carcinoma
HCV	Hepatitis C viruses
HRP	Horseradish peroxidase
iCCA	Intrahepatic cholangiocarcinoma
IHC	Immunohistochemistry
KCs	Kupffer cells
LMs	Liver macrophages
LPS	Lipopolysaccharides
LSM	Laser scanning microscope
MHC	Major histocompatibility complex
MDMs	Monocyte-derived macrophages
NAFLD	Non-alcoholic fatty liver disease
NKC	Natural killer cells
NO	Nitric oxide
NPCs	Non-parenchymal cells
PBS	Phosphate-buffered saline
pCCA	Perihilar cholangiocarcinoma
PD-L1	Programmed cell death ligand-1
PFA	Paraformaldehyde
PGE2	Prostaglandin E2
PHH	Primary human hepatocytes
PLD	Polycystic liver disease
PMA	Phorbol-12-myristat-13-acetat
PNG	Portable Network Graphic
PVE	Portal vein embolization
ROIs	Reactive oxygen intermediates
ROS	Reactive oxygen species
RT	Room temperature
SD	Standard deviation
SDS	Sodium dodecyl sulfate
TBS	Tris-buffered saline
TIFF	Tagged Image File Format
TLR	Toll-like receptor
s/p	status post
XTT	2,3-Bis-(2-Methoxy-4-Nitro-5-Sulfophenyl)-2H-Tetrazolium-5-Carboxanilide
WSI	Whole Slide Images
