# Supplementary material for: In Vivo and In Vitro Characterization of Primary Human Liver Macrophages and Their Inflammatory State
Source: Biomedicines. 2021 Apr 9;9(4):406. doi: 10.3390/biomedicines9040406 (PMC8070551; doi:10.3390/biomedicines9040406)
Supplement: Supplementary file 1 [file biomedicines-09-00406-s001.pdf]

biomedicines

MDPI

Supplementary Materials

Table S1. Detailed donor data for liver tissue samples used for immunohistochemical stainings of the targets CD68, CD80 and CD163. The table includes age, sex and disease as well as co-morbidities, CTx and drugs, BMI and relevant laboratory values.

Donor ID	Age, Sex	Disease	Co-morbidities	CTx [1] and drugs	BMI [2]	CRP [3] [mg/l]	ALAT [4] [µkat/l]	ASAT [5] [µkat/l]	GGT [6] [µkat/l]	Bilirubin, Total [µmol/l]
**D01**	38, f [7]	CRLM [8]	neurodermitis		24.3	0.69	0.4	0.46	0.27	4.9
**D02**	74, f	CRLM	chronic kidney failure, diabetes, arterial hypertension	neoadjuvant and adjuvant CTx	25.2	0.96	0.25	0.35	0.4	5.6
**D03**	50, m [9]	CRLM	s/p [10] SIRT [11], s/p in situ split, NAFLD [12]/AFLD [13]	neoadjuvant and adjuvant CTx	31.2		6.08	5.64	0.76	25.8
**D04**	46, f	iCCA [14]	low cholestasis, ulcerative colitis, gastritis	anti-inflammatory drugs (Azathioprin, Prednisolon, Salofalk)	33.5	38.7	0.27	0.41	2.08	3.5
**D05**	77, f	HCC [15]	low cholestasis, arterial hypertension, peripheral arterial occlusive disease, s/p melanoma		19.3	3.51	0.4	0.53	0.34	6.9
**D06**	77, f	pCCA [16], with infiltration of liver tissue	moderate cholestasis, pruritus / icterus, arterial hypertension, bronchial asthma		21.5	8.1	1.58	1.03	3.69	74.9
**D07**	65, f	pCCA, Bismuth IV with infiltration of liver tissue	moderate cholestasis and cholangitis, mild cholecystitis hyperlipidemia,hyper-uricemia, arterial hypertension, transient ischemic attack, depression		31.5	22.4	0.34	0.83	14.92	17.8
**D08**	75, m	pCCA, Bismuth III with infiltration of the portal vein bifurcation	moderate cholestasis, diabetes, arterial hypertension, atrial fibrillation, diverticulosis		31.0	3.18	0.72	0.63	2.42	19.5
**D09**	59, m	HCC, cirrhosis	diabetes, arterial hypertension, urothelial carcinoma,		29.5	33.21	0.25	0.34	1.7	4.4

			open wound treatment for Charcot foot, obstructive sleep apnea						
**D10**	79, f	HCC, Child A cirrhosis	diabetes, arterial hypertension, osteoporosis, s/p hepatitis B	27.6	3.51	0.58	0.75	1.72	9.1
**D11**	69, m	Bilioma (infected)	diabetes, arterial hypertension, s/p HCC in Child A cirrhosis, NAFLD/AFLD	39.2	20.62	0.73	0.87	10.92	11.5
**D12**	24, f	Adenoma	arterial hypertension, bronchial asthma	39.6	25.63	0.45	0.3	0.82	3.8
**D13**	41, m	FNH [17]	multiple myeloma, ulcerative colitis, chronic kidney failure, s/p stem cell transplantation	26.0	1.04	0.45	0.45	0.52	8.6
**D14**	26, f	Adenoma	diabetes, arterial hypertension, polycystic ovary syndrome, Graves' disease, s/p hepatitis B/C	33.9	6.55	1.64	1.15	2.48	5.2
**D15**	55, f	Hemangioma	arterial hypertension	23.6	0.84	0.43	0.46	1.02	11

[1] CTx: chemotherapy, [2] BMI: body mass index, [3] CRP: C-reactive protein, [4] ALAT: alanine aminotransferase, [5] ASAT: aspartate aminotransferase, [6] GGT: gamma-glutamyltransferase, [7] f: female, [8] CRLM: colorectal liver metastasis, [9] m: male, [10] s/p: status post, [11] SIRT: selective internal radiation therapy, [12] NAFLD: non-alcoholic fatty liver disease, [13] AFLD: alcoholic fatty liver disease, [14] iCCA: intrahepatic cholangiocarcinoma, [15] HCC: hepatocellular carcinoma, [16] pCCA: perihilar cholangiocarcinoma, [17] FNH: focal nodular hyperplasia

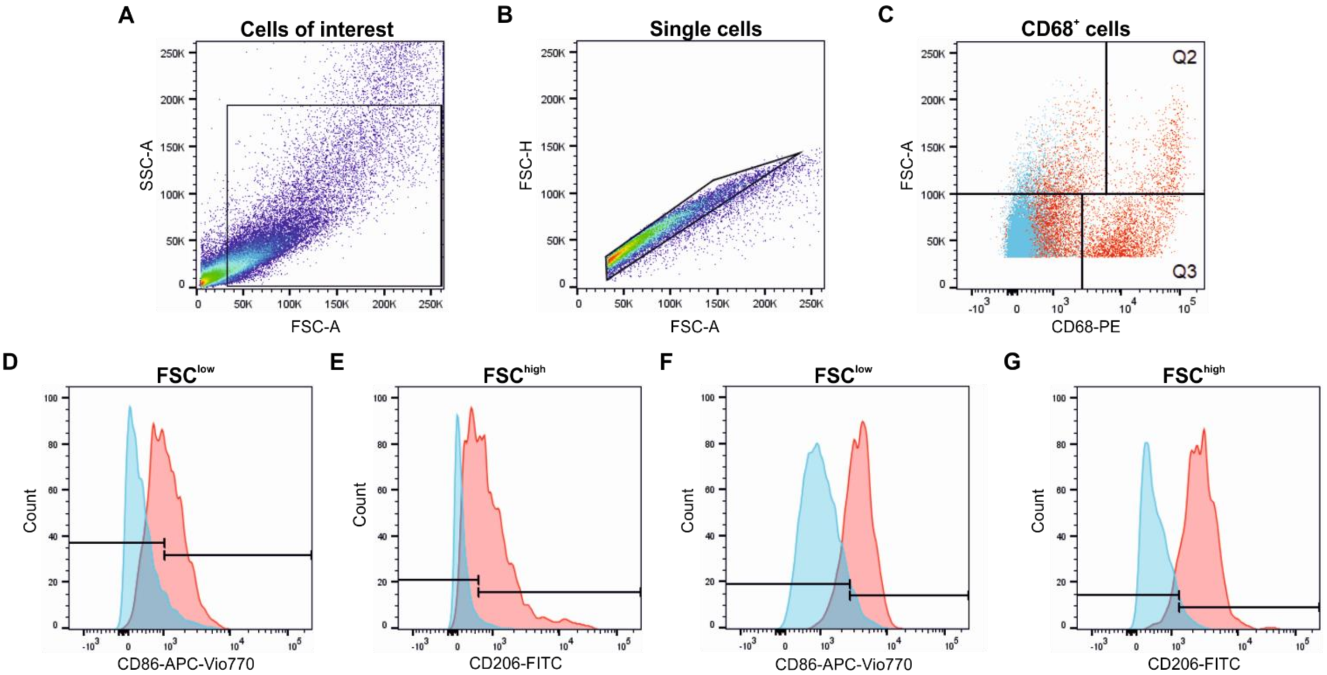

Figure S1. Gating strategy for Flow cytometric analysis. Exemplary representation using cells from D30. (**A**) First, cells of interest and from this (**B**) single cells were gated. In the following images the stained sample (red) is shown compared to the corresponding FMO control (blue). (**C**) We identified two CD68+ subpopulations with different FSC due to different cell sizes (Q2: FSC^{high}, Q3: FSC^{low}). These populations were analyzed separately for their expression of (**D**,**F**) CD86 and (**E**,**G**) CD206. In histograms, cell number was normalized to the modal value. (FMO: fluorescence minus one, FSC: forward scatter, SSC: side scatter)

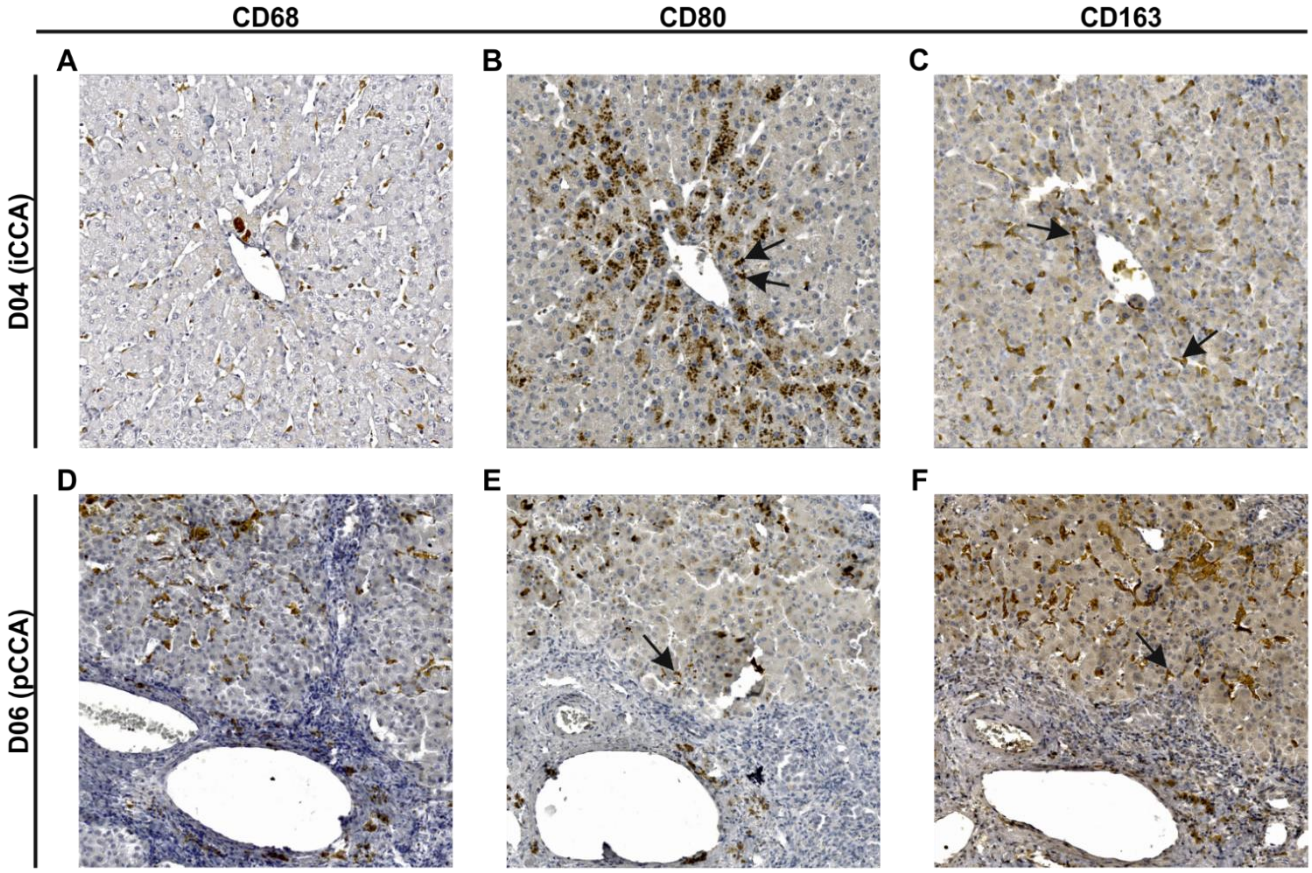

Figure S2. Determination of LM subpopulations in human liver tissue sections by analyzing parallel immunohistochemical stainings. Human liver tissue samples were investigated by immunohistochemical stainings for general macrophages (CD68), pro-inflammatory (CD80) and anti-inflammatory macrophages (CD163). Detection was performed using HRP-coupled secondary antibodies and visualization was done using DAB. LM subpopulations were determined by locating and comparing the same tissue regions in all stainings of one donor. Representative pictures of these are shown here for illustration purposes. (**A–C**) Images originate from a patient with iCCA (D04) and (**D–F**) from a patient with pCCA (D06) showing parallel stainings for CD68, CD80 and CD163. The arrows in (**B+E**) indicate CD68-/CD80+ cells, whereas arrows in (**C+F**) indicate CD68-/CD163+ cells. Images were taken using a Slide Scanner (AxioScan Z1, Carl Zeiss, Oberkochen, Germany) at 20× magnification. The whole tissue scans of all donors and immunohistochemical stainings are accessible by using the platform 'LiSyM SEEK' (https://seek.lisym.org/investigations/25 (accessed on April 8th 2021)). (iCCA: intrahepatic cholangiocarcinoma, LM: liver macrophage, pCCA: perihilar cholangiocarcinoma)

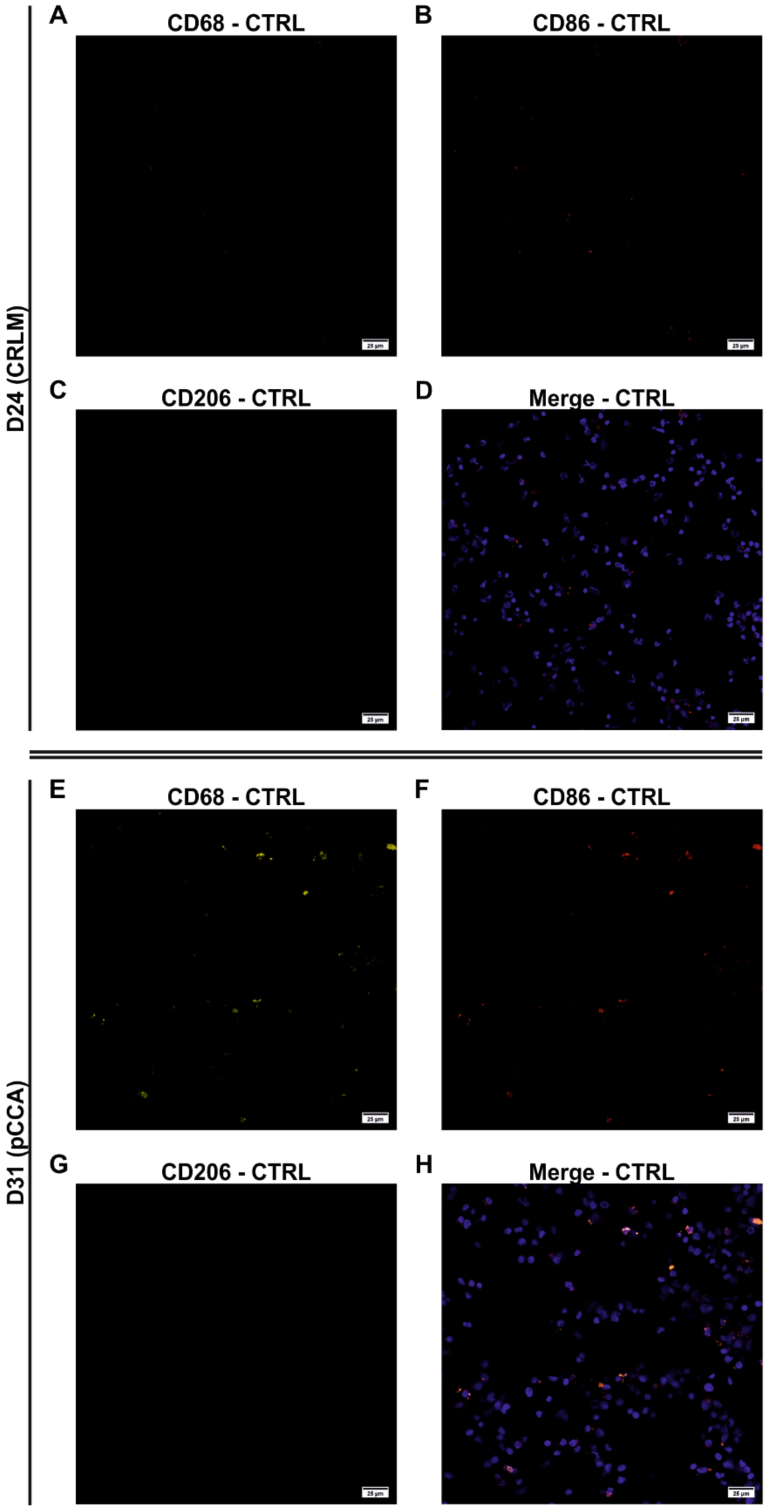

Figure S3. Autofluorescence controls for the qualitative evaluation of individual LM subpopulations from D24 and D31 (detailed donor data: Table 2). Confocal laser scanning microscopy of adherent and fluorescence-stained human hepatic macrophages was used to assess individual subpopulations. Autofluorescence controls shown here were treated like the other cultures without using antibodies. The cells from D24 with CRLM and D31 with pCCA were investigated regarding the marker (**A**,**E**) CD68 for general macrophages (yellow), (**B**,**F**) CD86 for pro-inflammatory (red) and (**C**,**G**) CD206 for anti-inflammatory macrophages (green). (**D**,**H**) The merged images show all markers simultaneously as well as the cell nuclei stained with Hoechst 33342

(blue). Images were taken using a laser scanning microscope (LSM 700, Carl Zeiss). (CRLM: colorectal liver metastasis, CTRL: control, LM: liver macrophages, pCCA: perihilar cholangiocarcinoma).

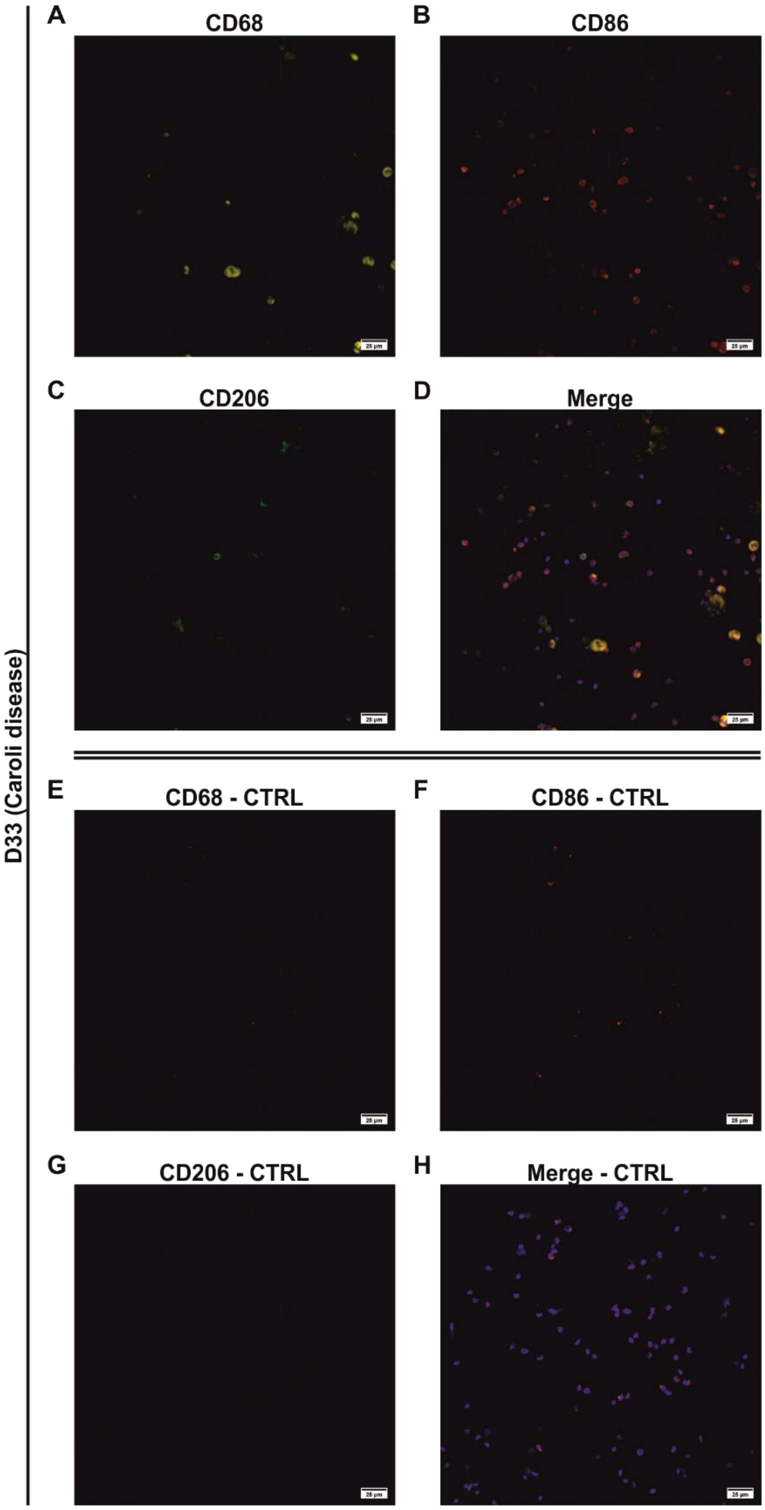

Figure S4. Qualitative evaluation of individual LM subpopulations from D33 (detailed donor data: Table 2). Confocal laser scanning microscopy of adherent and fluorescence-stained human hepatic macrophages was used to assess individual subpopulations. The cells from D33 with Caroli disease were investigated regarding the marker (**A**) CD68 for general macrophages (yellow), (**B**) CD86 for pro-inflammatory (red) and (**C**) CD206 for anti-inflammatory macrophages (green). (**D**) The merged images show all markers simultaneously as well as the cell nuclei stained with Hoechst 33342 (blue). (**E**–**H**) Autofluorescence controls shown here were treated like the other cultures without using antibodies. Images were taken using a laser scanning microscope (LSM 700, Carl Zeiss). (CTRL: control, LM: liver macrophages)

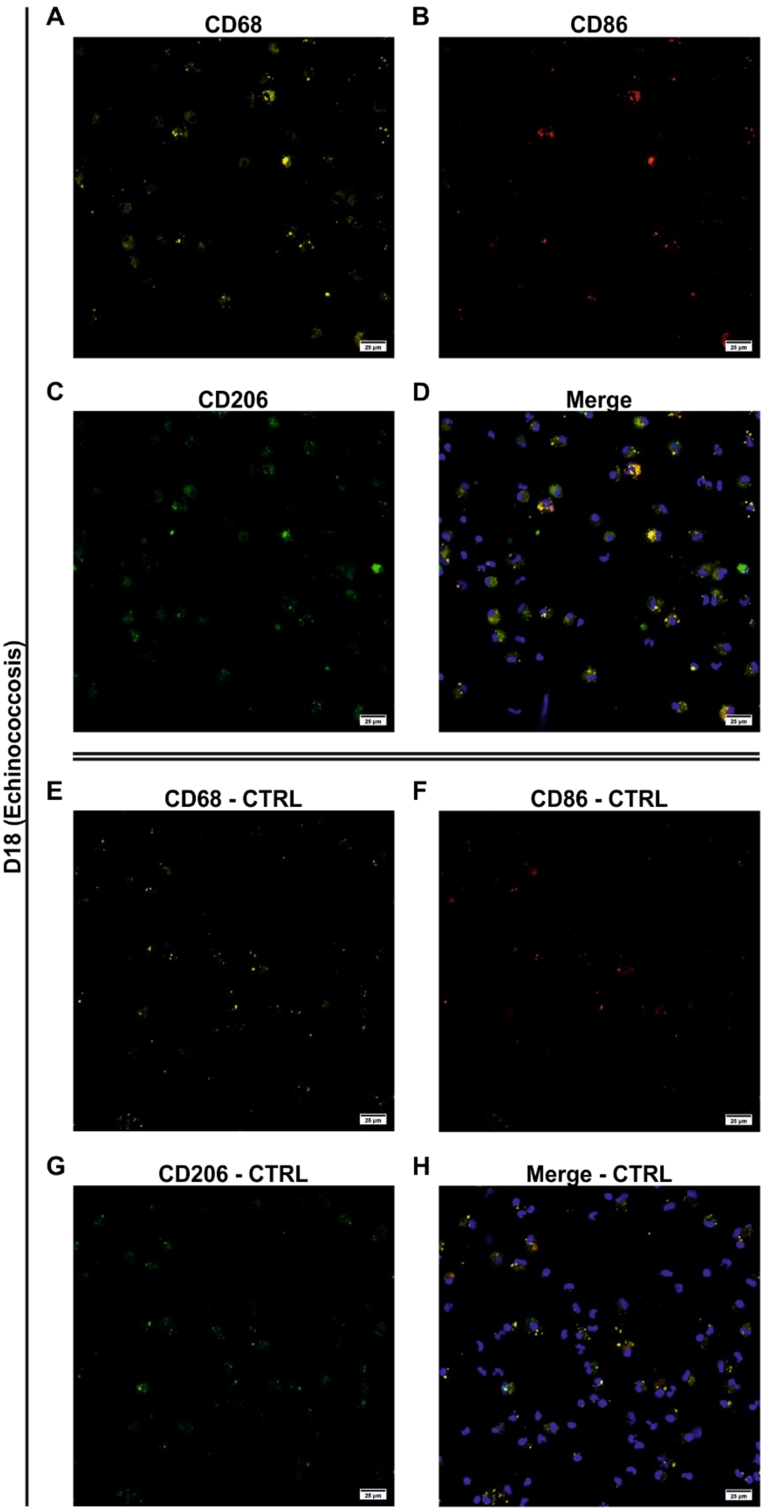

Figure S5. Qualitative evaluation of individual LM subpopulations from D18 (detailed donor data: Table 2). Confocal laser scanning microscopy of adherent and fluorescence-stained human hepatic macrophages was used to assess individual subpopulations. The cells from D18 with echinococcosis were investigated regarding the marker (**A**) CD68 for general macrophages (yellow), (**B**) CD86 for pro-inflammatory (red) and (**C**) CD206 for anti-inflammatory macrophages (green). (**D**) The merged images show all markers simultaneously as well as the cell nuclei stained with Hoechst 33342 (blue). (**E–H**) Autofluorescence controls shown here were treated like the other cultures without using antibodies. Images were taken using a laser scanning microscope (LSM 700, Carl Zeiss). (CTRL: control, LM: liver macrophages)

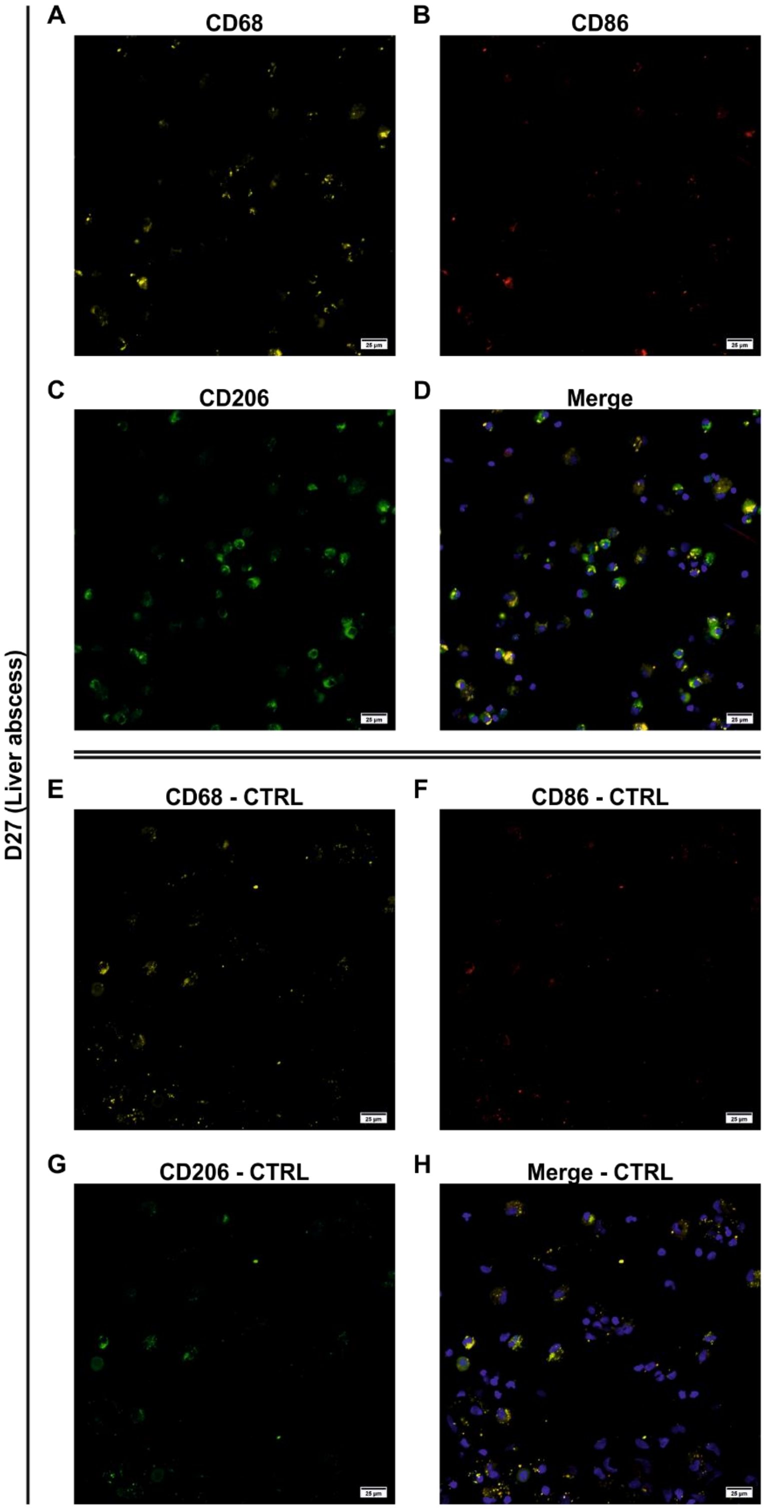

Figure S6. Qualitative evaluation of individual LM subpopulations from D27 (detailed donor data: Table 2). Confocal laser scanning microscopy of adherent and fluorescence-stained human hepatic macrophages was used to assess individual subpopulations. The cells from D27 with liver abscess were investigated regarding the marker (**A**) CD68 for general macrophages (yellow), (**B**) CD86 for pro-inflammatory (red) and (**C**) CD206 for anti-inflammatory macrophages (green). (**D**) The merged images show all markers simultaneously as well as the cell nuclei stained with Hoechst 33342 (blue). (**E–H**) Autofluorescence controls shown here were treated like the other cultures without using antibodies. Images were taken using a laser scanning microscope (LSM 700, Carl Zeiss). (CTRL: control, LM: liver macrophages).
